# NanoPARE: parallel analysis of RNA 5′ ends from low-input RNA

**DOI:** 10.1101/gr.239202.118

**Published:** 2018-12

**Authors:** Michael A. Schon, Max J. Kellner, Alexandra Plotnikova, Falko Hofmann, Michael D. Nodine

**Affiliations:** 1 Gregor Mendel Institute (GMI), Austrian Academy of Sciences, Vienna Biocenter (VBC), 1030 Vienna, Austria

## Abstract

Diverse RNA 5′ ends are generated through both transcriptional and post-transcriptional processes. These important modes of gene regulation often vary across cell types and can contribute to the diversification of transcriptomes and thus cellular differentiation. Therefore, the identification of primary and processed 5′ ends of RNAs is important for their functional characterization. Methods have been developed to profile either RNA 5′ ends from primary transcripts or the products of RNA degradation genome-wide. However, these approaches either require high amounts of starting RNA or are performed in the absence of paired gene-body mRNA-seq data. This limits current efforts in RNA 5′ end annotation to whole tissues and can prevent accurate RNA 5′ end classification due to biases in the data sets. To enable the accurate identification and precise classification of RNA 5′ ends from standard and low-input RNA, we developed a next-generation sequencing-based method called nanoPARE and associated software. By integrating RNA 5′ end information from nanoPARE with gene-body mRNA-seq data from the same RNA sample, our method enables the identification of transcription start sites at single-nucleotide resolution from single-cell levels of total RNA, as well as small RNA-mediated cleavage events from at least 10,000-fold less total RNA compared to conventional approaches. NanoPARE can therefore be used to accurately profile transcription start sites, noncapped RNA 5′ ends, and small RNA targeting events from individual tissue types. As a proof-of-principle, we utilized nanoPARE to improve *Arabidopsis thaliana* RNA 5′ end annotations and quantify microRNA-mediated cleavage events across five different flower tissues.

Diverse RNA 5′ ends are generated during and after transcription as the result of a variety of gene regulatory functions. Alternative transcription start sites (TSSs) can generate RNA isoforms that differentially impact cellular activities. Alternative TSSs have also been demonstrated to affect downstream translation and protein function through inclusion or exclusion of regulatory N-terminal peptides such as upstream open reading frames or protein localization sequences (Haberle et al. 2014; Ushijima et al. 2017; Cheng et al. 2018). Post-transcriptional maturation of noncoding RNAs such as those involved in splicing (snoRNA) or translation (rRNAs, tRNAs) also generates diverse RNA 5′ ends (Wang et al. 1988; Filipowicz and Pogačić 2002; Granneman et al. 2011; Henras et al. 2015). Moreover, small regulatory RNAs such as microRNAs (miRNAs) and small interfering RNAs (siRNAs) can mediate endonucleolytic cleavage of target RNAs and are important regulators of development, genome stability, and defense (Bartel 2004; Borges and Martienssen 2015). Therefore, the identification of RNA 5′ ends derived from transcriptional and post-transcriptional processes is important for the functional characterization of RNA molecules.

Next-generation sequencing (NGS)-based methods have recently been used to identify RNA 5′ ends genome-wide. For example, TSS profiling using cap analysis of gene expression (CAGE) led to the annotation of TSSs from polyadenylated mRNA and long noncoding RNA (Andersson et al. 2014; Hon et al. 2017). TSS profiling has also provided fundamental insights into how RNA isoforms with different 5′ ends modulate gene function, as recently demonstrated in *Arabidopsis thaliana* (*Arabidopsis*) where the phytochrome photoreceptor regulates alternative promoter usage in a light-dependent manner that ultimately leads to gene products with distinct functions and subcellular localization (Ushijima et al. 2017). Methods referred to as PARE (parallel analysis of RNA ends), or degradome, sequencing enrich for 5′ monophosphorylated RNAs, which include small RNA (sRNA)-mediated cleavage products. PARE methods therefore enable the genome-wide profiling of sRNA target sites and have been instrumental in characterizing the mechanistic basis of sRNA-mediated developmental and physiological processes (Addo-Quaye et al. 2008; German et al. 2008; Gregory et al. 2008).

Cell-type–specific TSSs and sRNA-mediated cleavage events contribute to the diversification of cellular transcriptomes and thus can impact cellular differentiation (Kidner and Martienssen 2004; Williams et al. 2005; Carlsbecker et al. 2010; Knauer et al. 2013; Miyashima et al. 2013; Zhou et al. 2015; Karlsson et al. 2017). However, due to technical limitations, RNA 5′ ends have traditionally been profiled on whole organisms or tissues composed of multiple cell types, and thus RNA 5′ ends that exist in specific cell types will be depleted in the corresponding final data sets. Recently, methods utilizing reverse transcriptase template-switching have been developed to profile TSSs from the low amounts of RNA obtainable from specific cell types and individual cells (Islam et al. 2011; Arguel et al. 2017; Cole et al. 2018). However, it remains a challenge to identify and confidently assign bona fide TSSs to their corresponding genes due to technical artifacts and variable performance between protocols (Cocquet et al. 2006; Tang et al. 2013; Adiconis et al. 2018). Moreover, PARE methods require high amounts of input RNA that limit their application to samples that can be collected in bulk.

## Results

### RNA 5′ end enrichment from low-input RNA samples

**Figure 1.**
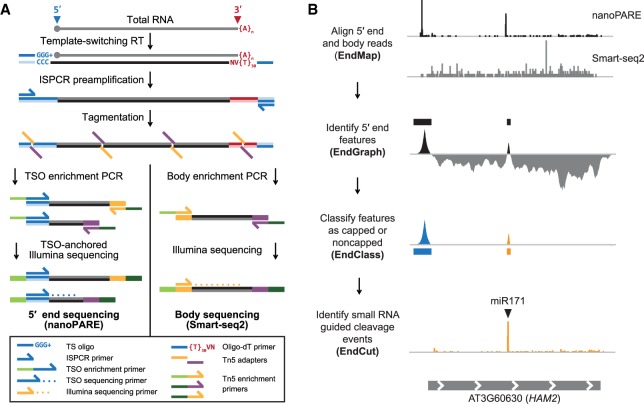
Workflow of nanoPARE and EndGraph. (*A*) Diagram of the nanoPARE protocol, which enables construction of a stranded 5′ end library (*left*) in parallel with a nonstranded transcript body library (Smart-seq2, (Picelli et al. 2013) from the same RNA sample. All oligonucleotides are labeled in the legend *below*. (*B*) Workflow of the nanoPARE data analysis pipeline for identifying distinct capped and noncapped 5′ end features from a paired nanoPARE and Smart-seq2 sequencing library. Diagram represents the output of each step, using *HAM2* as an example.

To profile RNA 5′ ends genome-wide from low amounts of total RNA, we developed an NGS-based method called nanoPARE (parallel analysis of RNA 5′ ends from low-input RNA) (Fig. 1A,B; see Methods). First, we followed the Smart-seq2 protocol through cDNA preamplification to produce full-length cDNAs with template-switching oligonucleotide (TSO) sequences at their 5′ ends (Picelli et al. 2013). Tn5 transposase was then used to fragment the cDNA and ligate adapters for NGS library preparation (Ramsköld et al. 2012). To selectively amplify the 5′ ends of the cDNAs, we performed PCR on the tagmented products using primers complementary to TSOs and inserted transposase adapter sequences. The resulting amplicons were then used for final PCR amplification with indexed Illumina-adapter primers for next-generation sequencing. Additionally, tagmented products corresponding to nonterminal, or body, regions of transcripts were amplified according to the Smart-seq2 method (Picelli et al. 2013). By combining the 5′ end and body sequence information from the cDNA of a single sample, the 5′ ends of RNA can be precisely identified at single-nucleotide resolution as demonstrated below.

### Identification of capped and noncapped 5′ end features

Template switching readily occurs at the 5′ ends of RNA templates with or without 7-methylguanosine (m7G) cap structures (Cloonan et al. 2008; Harbers et al. 2013). We reasoned that template switching could be used to identify RNA 5′ end features genome-wide regardless of their cap structure. However, spurious 5′ ends could be produced by a variety of technical artifacts, including random fragmentation of RNA in vitro, stalling of the reverse transcriptase enzyme, PCR amplification bias, or “strand invasion” by the template-switching oligo (Cocquet et al. 2006; Tang et al. 2013), which makes it difficult to distinguish biological signal from noise. A major source of nonrandom bias is internal template switching at sites complementary to the TSO 3′ end, and signal at these sites can be removed in silico (Tang et al. 2013). For other sources of noise, we developed a “scaling factor” for comparing nanoPARE libraries to a counterpart Smart-seq2 library from the same cDNA (see Methods). The scaling factor estimates the expected ratio of 5′ end containing cDNA fragments to gene body fragments after tagmentation with the assumption that all RNA is full-length. After applying this scaling factor, genomic regions are identified that produce more terminal signal than nonterminal signal, and these regions are isolated as 5′ end features using the software EndGraph (see Methods).

**Figure 2.**
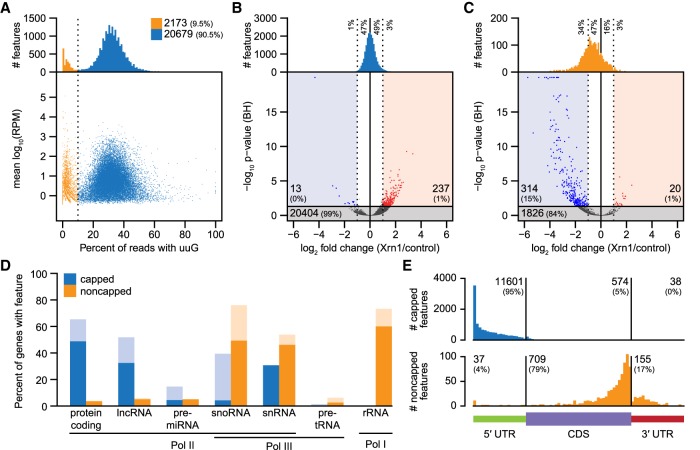
Identification of capped and noncapped 5′ end features with EndGraph. (*A*) RNA 5′ end features identified from 5 ng of floral bud total RNA, distributed by the proportion of nanoPARE reads containing an upstream untemplated guanosine (uuG). The vertical line separates putative noncapped features (low-uuG, orange) from putative capped features (high-uuG, blue). (*B*) Volcano plot of the change in read abundance for putative capped features after digestion with Xrn1 exonuclease. Bar plots depict the distribution of all capped features by fold change versus control. Dotted lines delimit a twofold change in feature abundance. Log_2_ fold change and Benjamini-Hochberg adjusted *P*-values (BH) were calculated by DESeq2. Horizontal line demarcates an adjusted *P*-value of 0.05. (*C*) Volcano plot as in *B* for putative noncapped features. (*D*) Capped and noncapped features overlapping TAIR10 genes classified by gene type. Lighter bars include features up to 500 nt upstream of the annotation. (*E*) Positional distribution of capped (*top*) and noncapped (*bottom*) features that overlap protein-coding genes.

We applied EndGraph to a paired collection of nanoPARE/Smart-seq2 libraries prepared from 5 ng of total RNA isolated from *Arabidopsis* floral buds in biological triplicate and reproducibly identified a total of 22,852 5′ end features from polyadenylated RNA in at least two of the three biological replicates (Supplemental Data S1, S2). Reverse transcription produces untemplated cytosines at the template 5′ terminus that can base-pair with a m7G cap to yield an untemplated upstream guanosine (uuG) in the cDNA between the TSO and genome-matching sequence (Cumbie et al. 2015; de Rie et al. 2017). These uuGs can be used to filter 5′ end data produced by template-switching protocols and isolate 5′-capped transcription start sites (Cumbie et al. 2015). Indeed, uuG consistently appeared in roughly 15% of nanoPARE reads per library, occurring from four to 10 times more frequently than other nucleotides (Supplemental Fig. S1). We analyzed the total proportion of reads in all 5′ end features that contained uuG, and we could observe a striking bimodal distribution, with most 5′ end features (20,679; 90.5%) containing >10% uuG reads (Fig. 2A). We hypothesized that this bimodal distribution indicated two populations of RNA 5′ ends: high-uuG “capped” features that contain an m7G cap, and low-uuG “noncapped” features that contain alternative 5′ end structures, predominantly monophosphates. The yeast Xrn1 exoribonuclease specifically degrades 5′-monophosphorylated RNA in the 5′-to-3′ direction, leaving capped RNA intact (Nagarajan et al. 2013). We performed nanoPARE on the same total RNA samples after subjecting them to exonucleolytic digestion by Xrn1 and found that, as expected, nearly all capped features showed no significant change in abundance after Xrn1 digestion (Fig. 2B). In contrast, a global decrease was observed in the abundance of putative noncapped features after Xrn1 digestion, with a mean reduction of 19.5%, and a statistically significant reduction in 314 of 2173 noncapped features (14.5%) (Fig. 2C).

### Genomic distributions of capped and noncapped 5′ features

Capped and noncapped features have distinct distributions in the genome. Capped features have an even distribution across the noncentromeric region of the nuclear genome, whereas noncapped features are highly concentrated on the mitochondrial and chloroplast genomes and more sparsely dispersed in the nuclear genome (Supplemental Fig. S2). The 5′ ends of chloroplast and mitochondrial RNA do not possess m7G caps (Grohmann et al. 1978; Monde et al. 2000; Legen et al. 2002). Accordingly, the 5′ features mapping to chloroplasts and mitochondria were classified as 94% and 95% noncapped, respectively. Moreover, capped and noncapped features localized to different gene types (Fig. 2D). Consistent with caps being associated only with RNA polymerase II (Pol II) transcripts, nanoPARE identified predominantly capped features for mRNA, long noncoding RNA, and primary microRNA (Supplemental Fig. S3). Several noncapped features mapping to pre-tRNA and rRNA loci, which are not transcribed by Pol II, could also be identified (Supplemental Figs. S4, S5). While these products normally do not possess a poly(A) tail, a subset will be transiently polyadenylated by the TRAMP complex prior to their degradation (Hopper et al. 2010), and nanoPARE may be detecting this subset. Even within a class of RNA, the behavior of capped and noncapped features is quite distinct. For example, a majority of snoRNAs in *Arabidopsis* are transcribed in tandem arrays of two or more species from a single Pol II precursor (Dieci et al. 2009). EndGraph identified both a noncapped feature at the mature 5′ terminus and a capped feature upstream of many annotated snoRNAs (Fig. 2D). Several primary snoRNAs contained additional noncapped features not predicted by TAIR10 annotations, and these are supported by genome-wide profiling of noncoding RNAs (Supplemental Fig. S3; Wang et al. 2014).

Protein-coding mRNA also displays a distinct distribution of capped and noncapped 5′ end features. Among capped features overlapping nuclear protein-coding genes, 95% mapped to the annotated 5′ UTRs (Fig. 2E). Noncapped features showed an opposite trend, accumulating closer to the 3′ termini of protein-coding genes, particularly just upstream of the stop codon. This pattern closely resembles the reported pattern of cotranslational decay detected in PARE data, which is mediated by the major cytoplasmic exonuclease EXORIBONUCLEASE4 (XRN4) (Hou et al. 2016; Yu et al. 2016). To test whether we detect the steady state by-products of XRN4 digestion with nanoPARE, we generated nanoPARE and Smart-seq2 libraries for floral buds of *xrn4-5* mutants and performed de novo 5′ feature identification. All features identified in wild-type floral buds (with or without Xrn1 digestion) and/or *xrn4-5* floral buds were combined to produce a unified set of 5′ end features (Supplemental Data S2). Because XRN4 is restricted to the cytoplasm (Kastenmayer and Green 2000), noncytoplasmic 5′-monophosphorylated ends should not be increased in *xrn4-5* loss-of-function mutants. As predicted, noncytoplasmic 5′ end features associated with mature or primary noncoding RNAs including nuclear 3′ cleavage products of pri-snoRNA, pre-miRNA, as well as mitochondrial and chloroplast pri-tRNA (Supplemental Data S3), were not increased in *xrn4-5* floral buds relative to wild type. In contrast, these features were sensitive to Xrn1 digestion in vitro (Supplemental Fig. S6).

Consistent with wild-type XRN4 digesting full-length decapped mRNA, noncapped features upstream and adjacent to stop codons were globally decreased in relative abundance in *xrn4-5* mutants, concomitant with a relative increase in reads contained by capped features (Supplemental Fig. S7). Together, these trends predict an increase in full-length transcripts with 5′-monophosphates in *xrn4* mutants. Indeed, the accumulation of full-length decapped mRNA has been reported for some transcripts in *xrn4* mutants (Gregory et al. 2008). We reanalyzed public PARE data from wild-type and *xrn4-5* inflorescences (German et al. 2008) and found a global average increase by more than threefold in the proportion of 5′ monophosphorylated RNA ends mapping to all capped features defined by nanoPARE (Supplemental Fig. S7). Overall, the capped and noncapped 5′ features identified with nanoPARE support the existing model of XRN4 as a general RNA decay factor that acts downstream from decapping.

### TSS characterization

**Figure 3.**
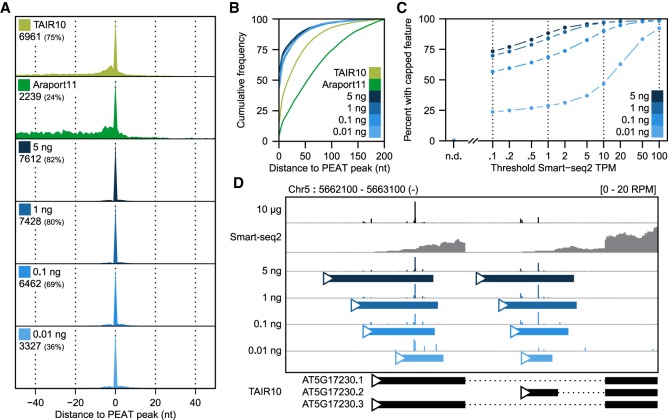
Sensitive low-input transcription start site detection with nanoPARE. (*A*) Recall of capped peaks identified with PEAT (Morton et al. 2014) in two *Arabidopsis* reference annotations (TAIR10 and Araport11) and in nanoPARE features detected from a dilution series of total RNA input. Numbers indicate how many PEAT peaks have a 5′ end feature within 50 bp in the test data set. (*B*) Cumulative frequency distribution of positional error for all 5′ features within 200 nt of a PEAT peak. (*C*) Sensitivity of nanoPARE in detecting capped 5′ features for nuclear protein-coding genes as a function of their abundance measured by Smart-seq2. Points indicate the percent of transcripts above the given threshold abundance (in transcripts per million, TPM) that contain a capped feature identified in at least two of three biological replicates. (*D*) Integrated Genomics Viewer (IGV) browser image of nanoPARE reads from the dilution series mapping to two transcription start sites of the *PSY* locus. *y*-axis shows mean reads per million (RPM) across three biological replicates for each dilution. Solid colored bars mark capped features identified by EndGraph in each dilution.

To test the reproducibility of nanoPARE to detect 5′ end features from low-input RNA, we generated nanoPARE libraries from a dilution series of the original floral bud total RNA in triplicate: 1 ng, 100 pg, and 10 pg of total RNA input, which is typically less than or equal to the amount of total RNA found in a single cell (Ramsköld et al. 2012; Brennecke et al. 2013). We performed de novo feature identification using the Smart-seq2 libraries from 5 ng of RNA as a background model. To assess the sensitivity of the method at recovering genuine capped transcription start sites, we compared the capped features of the dilution series to a set of 9326 transcription start sites identified by a cap-specific 5′ sequencing protocol (paired-end analysis of transcription start sites; PEAT) applied to whole *Arabidopsis* roots (Morton et al. 2014). As a baseline, we tested whether the *Arabidopsis* reference annotations, TAIR10 and Araport11, contained a transcript model with a 5′ end within 50 bp of a given PEAT peak. TAIR10 detected 75% of PEAT peaks under this definition (Fig. 3A). The more recent Araport11 annotations performed much worse at accurately detecting transcription start sites. Only 2239 (24%) of PEAT peaks fell within 50 bp of any transcript 5′ ends defined in Araport11, which is likely due to the systematic overextension of transcript UTRs during transcript model assembly. Despite the different tissue type and preparation method, nanoPARE outperformed both reference annotations at detecting experimentally validated transcription start sites, down to 1 ng of total RNA (Fig. 3A). Furthermore, when comparing the precision of peaks, all nanoPARE dilutions, including those generated from 10 pg of total RNA, had a higher likelihood of agreeing with the PEAT data on the exact nucleotide position of the transcription start site peak (Fig. 3A,B). Finally, we examined the sensitivity of the method by comparing identified capped features with the transcript abundance as measured by Smart-seq2 (Fig. 3C). Remarkably, nanoPARE reproducibly identified at least one capped feature overlapping 76.6% of all protein-coding transcripts detected at or above 0.1 transcripts per million (TPM) when libraries were generated from 5 ng of total RNA (18,295/23,900 genes). This value increased to 91.9% for transcripts detected at a threshold of 1 TPM (18,111/19,706 genes), and 99.0% for transcripts of at least 10 TPM (11,165/11,294 genes). In contrast, only 0.4% of transcripts not detected with Smart-seq2 (0 TPM, 20/5419 genes) were assigned a capped feature. Overall, the 5-ng, 1-ng, and 100-pg samples performed similarly well, especially for robustly detected transcripts. Sensitivity reduced substantially between 100 and 10 pg of total RNA without affecting the precision of the capped features identified (Fig. 3A,B). Multiple distinct TSSs could even be identified at all dilutions for certain highly expressed genes (Fig. 3D; Supplemental Data S2). Therefore, nanoPARE capped features represent genuine transcription start sites and can be used for transcription start site annotation with as little as 10 pg of input RNA.

### Detecting sRNA-mediated cleavage sites

**Figure 4.**
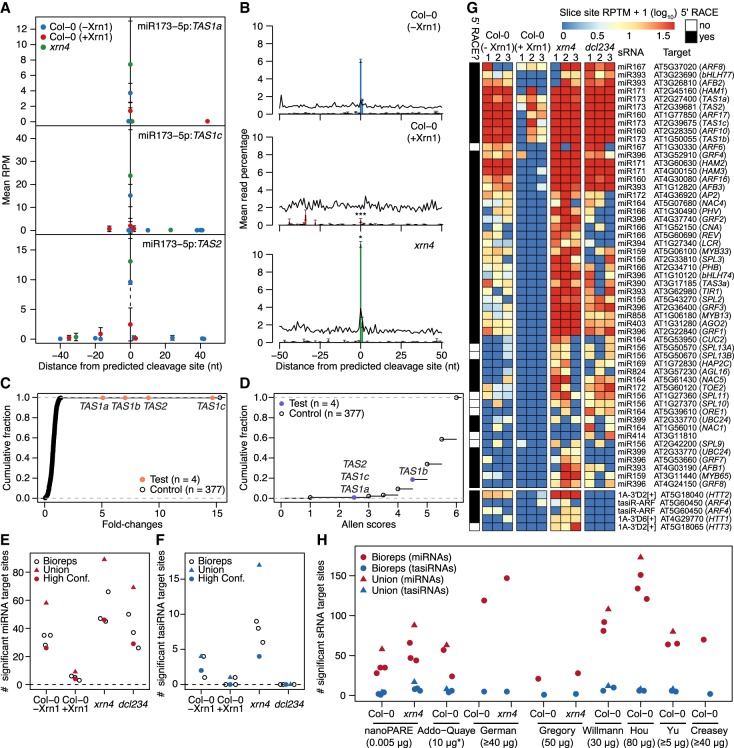
Detection of sRNA-mediated cleavage sites. (*A*) Scatter plot illustrating the number of nanoPARE read 5′ ends per million transcriptome-mapping reads within 50 nt of predicted miR173-5p–directed cleavage sites in *TAS1a* (*top*), *TAS1c* (*middle*), and *TAS2* (*bottom*) transcripts. Mean RPM values of three biological replicates are shown for libraries prepared from 5 ng of total RNA from wild-type (Col-0) floral buds either not incubated with Xrn1 (Col-0 [−Xrn1]) or incubated with Xrn1 (Col-0 [+Xrn1]), or *xrn4-5* mutant floral buds (*xrn4*). Error bars represent standard errors of the means. (*B*) Number of nanoPARE read 5′ ends mapping within 50 nt of miRNA cleavage sites significantly detected by EndCut (Benjamini-Hochberg adjusted *P*-values < 0.05) in Col-0 (−Xrn1) libraries are shown as bar charts of the percentage of the total number of nanoPARE reads detected for each transcript in libraries prepared from Col-0 (−Xrn1) (*top*), Col-0 (+Xrn1) (*middle*), and *xrn4* (*bottom*) samples. Percentages of all predicted miRNA cleavage sites are shown as line graphs. * and *** indicate that the mean number of reads at predicted cleavage sites are significantly different in Col-0 (−Xrn1) libraries compared to either Col-0 (+Xrn1) or *xrn4* libraries (*P*-values <0.05 and 0.001, respectively; one-tailed K-S tests). (*C*,*D*) Cumulative fractions of fold changes (*C*) and Allen scores (*D*) are shown for target sites predicted for either miR173-5p (test) or its randomized cohorts (control). (*E*,*F*) One-dimensional scatter plots illustrating the number of significant miRNA (*E*) or tasiRNA (*F*) target sites (Benjamini-Hochberg adjusted *P*-values < 0.05) detected in libraries prepared from Col-0 (−Xrn1), Col-0 (+Xrn1), *xrn4*, or *dcl234* samples. Values for individual biological replicates (bioreps), all detected sites (union), and significant interactions observed in at least 2/3 bioreps (High conf.) are shown. (*G*) Heat maps depicting the number of nanoPARE read 5′ ends per 10 million transcriptome-mapping reads (RPTM; log_10_) mapping to the high-confidence miRNA- (*top*) or tasiRNA- (*bottom*) directed cleavage sites denoted in panels *E* and *F*. Small RNA families and corresponding targets are indicated *beside* each row, and targets previously verified by 5′ RACE are annotated. (*H*) One-dimensional scatter plot showing the number of significant miRNA and tasiRNA target sites detected with EndGraph from nanoPARE libraries prepared from Col-0 or *xrn4* floral bud total RNA (nanoPARE) or published degradome/PARE libraries prepared from Col-0 or *xrn4* floral tissue total RNA. Published degradome/PARE libraries are indicated by the first author of the corresponding study: Addo-Quaye (Addo-Quaye et al. 2008), German (German et al. 2008), Gregory (Gregory et al. 2008), Willmann (Willmann et al. 2014), Hou (Hou et al. 2016), Yu (Yu et al. 2016), and Creasey (Creasey et al. 2014). The amounts of total input RNA (µg) used in each publication are indicated. The asterisk denotes that the Addo-Quaye samples were prepared from polyadenylated RNA instead of total RNA.

In plants, Argonaute-bound sRNAs recognize highly complementary 20- to 22-nt target sites and mediate target RNA cleavage precisely between the tenth and eleventh nucleotides of the sRNA-target duplex (Llave et al. 2002; Kasschau et al. 2003; Jones-Rhoades and Bartel 2004). Because nanoPARE reads map to the 5′ ends of noncapped transcripts (Fig. 2), we reasoned that the first position of nanoPARE reads should also be enriched precisely at sRNA target cleavage sites and thus allow their identification from low-input RNA samples. To test whether nanoPARE reads from libraries generated with low-input RNA samples were enriched at sRNA target cleavage sites, we examined predicted cleavage sites for either miRNAs or *trans*-acting siRNAs (tasiRNAs) in libraries prepared from 5 ng of total RNA isolated from floral buds. The 5′ ends of nanoPARE reads were enriched at cleavage sites pairing to highly complementary miRNAs, including those from previously characterized miRNA cleavage sites in wild-type (Col-0) flowers (Fig. 4A,B; Supplemental Figs. S8, S9). As expected for sRNA-directed cleavage products that are 5′ monophosphorylated, the number of nanoPARE reads at cleavage sites were reduced when RNA was incubated with the Xrn1 exoribonuclease prior to library generation. Conversely, nanoPARE read enrichment at cleavage sites was increased in *xrn4-5* exoribonuclease mutants which stabilize sRNA cleavage products (Fig. 4A,B; Supplemental Figs. S8, S9; Souret et al. 2004; German et al. 2008).

In addition to biological variation between tissues or genotypes, variability between nanoPARE/PARE libraries can also be largely due to technical differences in RNA quality and quantity, as well as library complexity and sequencing depth. Therefore, we developed software called EndCut that employs empirically determined null models from randomized versions of each sRNA to compute the likelihood that nanoPARE read 5′ ends are enriched at predicted target sites greater than expected by chance in each library (Fig. 4C,D; Supplemental Fig. S10; see Methods). EndCut uses two metrics to calculate this likelihood: the level of sRNA-target complementarity (Allen score) and the number of read 5′ ends at predicted cleavage sites divided by the maximum number detected at a single site within 20 or 50 nt of flanking transcribed regions (fold change).

To assess the validity of EndCut, we examined the proportions of nanoPARE read 5′ ends within and adjacent to miRNA cleavage sites determined to be significant in at least one of the nanoPARE libraries prepared from Col-0 floral bud RNA without Xrn1 treatment (Col-0 [−Xrn1]). As expected for miRNA cleavage sites, the number of nanoPARE reads at these sites was significantly decreased 9.7-fold upon Xrn1 treatment (Col-0 [+Xrn1]) or significantly increased 1.8-fold in *xrn4-5* mutants (*P*-values = 1.08 × 10^−26^ and 0.037, respectively; one-tailed K-S tests) (Fig. 4B). Moreover, we compared the number of significant cleavage sites identified by EndCut in Col-0 floral buds either treated or not treated with Xrn1 prior to library construction, as well as from *xrn4-5* and tasiRNA-deficient *dcl234* mutant floral buds (Gasciolli et al. 2005; Yoshikawa et al. 2005; Henderson et al. 2006; Howell et al. 2007). We identified 58 total miRNA target sites in Col-0 floral buds with a mean of 32.7 miRNA target sites among three biological replicates (Supplemental Data S4). The mean number of miRNA target sites identified upon Xrn1 treatment was significantly reduced sevenfold and significantly increased 1.6-fold in *xrn4-5* (*P*-values = 1.52 × 10^−3^ and 0.041, respectively; one-tailed *t*-tests) (Fig. 4E). We also identified 26 significant miRNA target cleavage sites in at least two biological replicates of Col-0, and these high-confidence sites had decreased and increased numbers of nanoPARE read 5′ ends in libraries from Xrn1-treated RNA and *xrn4-5* mutants, respectively (Fig. 4G).

In addition to miRNAs, we also detected four significant tasiRNA target sites in Col-0 (−Xrn1) floral buds, including two of which were detected in at least two biological replicates. Similar to what was observed for miRNA cleavage sites, tasiRNA cleavage sites significantly detected in Col-0 (−Xrn1) samples had reduced numbers of reads mapping to cleavage sites upon Xrn1 treatment (Fig. 4F,G; Supplemental Data S5). In contrast, both the number of significantly detected tasiRNA target sites and reads mapping to cleavage sites were increased in *xrn4-5* mutants (Fig. 4F,G). Importantly, whereas miRNA target sites significantly detected in Col-0 (−Xrn1) samples were generally unaffected in tasiRNA-deficient *dcl234* mutants, none of the corresponding tasiRNA target sites were significantly detected in *dcl234* mutants, and zero reads corresponding to these target sites were observed (Fig. 4E–G). Out of the 58 high-confidence miRNA and tasiRNA target sites detected in either Col-0, *xrn4-5*, or *dcl234*, 49 (84.5%) had been previously validated by a modified 5′ RACE technique (Supplemental Data S6), while five of the remaining nine have genetic or expression data indicating that they are sRNA targets (Wu et al. 2006, 2009; Wang et al. 2009; Nodine and Bartel 2010). Based on the above biochemical and genetic tests, EndCut enables the accurate identification of sRNA-mediated cleavage events from nanoPARE data generated with as low as 5 ng of total RNA.

PARE methods capture sRNA cleavage products through a series of adapter-RNA ligations, Type IIS restriction enzyme digestions, and PCR amplifications (Addo-Quaye et al. 2008; German et al. 2008; Gregory et al. 2008). Because EndCut utilizes empirically determined null models based on randomized sRNAs, it can also be used to mitigate the effects of technical biases in conventional PARE data sets and help identify high-confidence target sites. As a proof-of-principle, EndCut was applied to 15 publicly available degradome/PARE data sets generated from at least 10 µg of total RNA from wild-type (Col-0) or *xrn4-5* mutant floral tissues. The number of significant cleavage sites detected with EndCut varied between publicly available data sets, but more sRNA-mediated cleavage events were detected from most PARE libraries compared to nanoPARE libraries, indicating that PARE libraries detect a greater diversity of cleavage sites when the starting amount of total RNA is not limiting (Fig. 4H; Supplemental Data S7, S8).

### Tissue-specific miRNA-mediated cleavage sites

**Figure 5.**
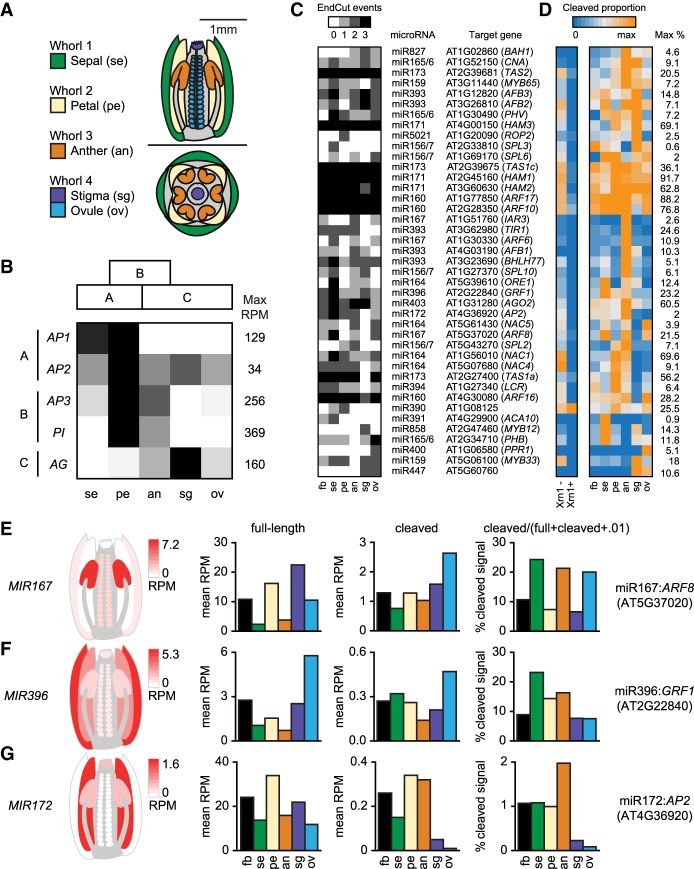
Tissue-specific miRNA-target interactions with nanoPARE. (*A*) Diagrams of a longitudinal section (*top*) and cross-section (*bottom*) of an *Arabidopsis* flower at the onset of anthesis. Tissue types isolated for nanoPARE libraries are color-coded as shown. (*B*) Relative expression of the five ABC model homeotic genes across the five tissue types in panel *A*. Each row is scaled from zero to the maximum observed reads per million of a gene's capped feature. Expected spatial distributions based on the ABC model are shown as blocks *above*. (*C*,*D*) Heat maps of 41 high-confidence miRNA cleavage sites detected by nanoPARE in whole flowers (fb) and individual tissue types illustrating either the number of biological replicates in which the cleavage site was significantly detected (EndCut events) (*C*) or the proportion of cleaved signal to total full-length and cleaved signal (*D*). Each row is scaled to the maximum proportion observed for that interaction, which is indicated on the *right*. (*E–G*) (*Left*) Heat maps of the summed primary transcript levels for three families of miRNA genes in flowers as measured by nanoPARE. Floral tissues match those labeled in panel *A*. (*Right*) Bar charts depicting the relative abundance of full-length RNA, truncated RNA with a 5′ end matching the miRNA cleavage site, and the proportion of cleaved RNA to the total cleaved and full-length signal, for the most strongly cleaved target of each of the three miRNA families to the *left*.

To test whether nanoPARE can detect small RNA-mediated cleavage sites that occur in specific tissue types, we applied the method to five different tissues dissected from whole flowers immediately after anthesis (Fig. 5A). The flower is comprised of four concentric whorls of tissues, which are specified by three transcription factor groups functioning in overlapping domains within the developing primordia. The coordinated action of these genes is described as the “ABC model” of flower development (Bowman et al. 1991b; Coen and Meyerowitz 1991). These transcription factors are known as group A, B, or C genes if they are expressed in the outer two whorls, middle two whorls, or inner two whorls of the developing flower, respectively. *Arabidopsis* possesses two A genes, two B genes, and a single C gene whose transcript spatial distributions are maintained through late flower development except for *APETALA2* (*AP2*) mRNA, which has been observed in all four whorls of mature flowers (Bowman et al. 1991a; Jack et al. 1992; Mandel et al. 1992; Goto and Meyerowitz 1994; Jofuku et al. 1994). Upon comparing the relative abundance of 5′ capped transcript ends, we observed that nanoPARE faithfully recapitulated the expected spatial transcript patterns of all five homeotic genes (Fig. 5B). Therefore, these data sets can be used to quantify tissue-specific variation in RNA abundance.

Tissue-specific variation of miRNA-target interactions on a genome-wide scale has not been reported in flowers, but individual studies indicate that miRNAs can suppress their targets in a tissue-specific manner (Wu et al. 2006; Wollmann et al. 2010; Liang et al. 2014). Upon performing nanoPARE on 10 ng of total RNA from either whole flowers or five individual floral tissues, we identified 41 miRNA target sites in at least two biological replicates of the same floral tissue (high-confidence sites) (Fig. 5C; Supplemental Data S4). While the target cleavage sites directed by three miRNAs (miR160, miR171, and miR173) are robustly detected across all tissues examined, over half of the high-confidence interactions were enriched in specific tissues. To better estimate differences in miRNA-guided cleavage activity, we calculated the proportion of nanoPARE signal at the cleavage site relative to the total cleaved and capped (full-length) signal for each gene (Fig. 5D; Supplemental Fig. S11). For most miRNA target interactions, the proportion of cleaved transcripts varied strongly between tissues. For example, we found that *AUXIN RESPONSE FACTOR 6* and *8* (*ARF6/8*) transcripts were preferentially cleaved in sepals, anthers, and ovules, which is consistent with both *MIR167* transcript levels as well as previous reports (Fig. 5E; Wu et al. 2006; Rubio-Somoza and Weigel 2013). miR396 spatially restricts seven transcripts encoding GROWTH-REGULATING FACTOR proteins (*GRF1/2/3/4/7/8/9*) to the developing carpel (Liang et al. 2014). Although only one target (*GRF1*) was identified in wild-type flowers, six were identified in *xrn4-5* mutant flowers, indicating that the cleavage products from this gene family are efficiently cleared from wild-type cells (Fig. 4G; Supplemental Fig. S11). Despite their transient nature, miR396-directed *GRF1* cleavage products accumulated to a higher proportion in noncarpel tissues than in stigmas or ovules (Fig. 5F), and full-length transcripts for the other targeted GRFs were restricted almost exclusively to ovules (Supplemental Fig. S11). Lastly, genetic data supports a model whereby miR172 represses *AP2* in whorl 3 to maintain stamen identity (Wollmann et al. 2010). Consistent with this model, we detected higher miR172 activity in anthers compared to other tissues (Fig. 5G). Because the tissue-enriched miRNA-guided cleavage events detected by nanoPARE are in good agreement with these experimentally supported examples, we conclude that nanoPARE can be used to detect sRNA-guided cleavage events in specific tissue types.

## Discussion

NanoPARE can accurately profile RNA 5′ ends genome-wide from low amounts of total RNA. Because TSSs partition the genome into transcribed and *cis*-regulatory regions, their accurate identification is critical for transcriptome assembly and prediction of regulatory binding sites. Moreover, TSS can vary among cell types, and thus their identification from low-input RNA samples can increase our understanding of diverse RNA processing events and corresponding functions. NanoPARE's integrative approach of combining RNA 5′ end enrichment and full-length Smart-seq2 data sets from the same sample enables TSS identification at single-nucleotide resolution from single-cell to standard levels of total RNA. Accordingly, we have improved current *Arabidopsis* TSS annotations using this technique. NanoPARE's low-input RNA requirements and the simplicity of the protocol should enable TSS annotation improvements in other eukaryotic species, as well as in rare tissues and individual cell types.

The identification of sRNA-directed cleavage targets is essential to understand the molecular basis of sRNA functions during cellular differentiation, physiology, and defense. Conventional PARE/degradome methods have been key technologies for characterizing the molecular basis of sRNA-mediated regulation but require high amounts of input RNA typically only obtainable from bulk samples. NanoPARE allows identification of sRNA-mediated cleavage products from at least 10,000-fold less input RNA compared to these conventional methods and thus can be applied to specific tissue types. As a case study, we utilized nanoPARE to quantify miRNA-mediated cleavage events across five different flower tissues. In addition to detecting the previously reported tissue-enriched activities of three miRNA families, nanoPARE also identified several novel tissue-enriched miRNA-guided cleavage events, indicating that it can be used to profile differential sRNA activities across tissue types.

Moreover, nanoPARE enables 5′ end RNA profiling from existing full-length Smart-seq2 libraries, which has become a commonly used single-cell sequencing method (Ziegenhain et al. 2017). Such resampling at the level of cDNA rather than tissue is unique and not possible with technologies such as CAGE, STRT-Seq, Tn5-Prime, and PARE. We therefore envision future applications of nanoPARE on both existing and new data sets for dissecting cell-type–specific transcriptional and post-transcriptional RNA regulatory mechanisms.

## Methods

### Plant material and growth

Wild-type and mutant (*xrn4-5*, *dcl234*) seeds were in Col-0 accession backgrounds and were grown in climate-controlled growth chambers with 20°C–22°C temperature and 16 h light/8 h dark cycle. The *dcl234* mutants were composed of *dcl2-1*, *dcl3-1*, and *dcl4-2* alleles (Henderson et al. 2006), and *xrn4-5* mutants were as previously described (Souret et al. 2004).

### RNA extraction

Total RNA was extracted from stage 12 floral buds using TRIzol (Life Technologies). Stage 13 flowers were collected in 1 mL of 500 µM DTSSP (3,3′-dithiobis-[sulfosuccinimidyl propionate]) (Thermo Fisher, Cat. #21578) with 1× PBS, pH 7.4, vacuum-infiltrated for 5 min, and incubated for 10 min. Individual floral tissues were dissected under a binocular microscope on a silanized slide and snap-frozen in liquid nitrogen. Tissues were then homogenized using Mixer Mill MM 400 (Retsch), and the resulting pellets were resuspended in 300 µL TRIzol (Life Technologies). Total RNA was extracted using a Direct-zol kit (ZymoResearch) according to the manufacturer's instructions. RNA integrity was assessed by a Fragment Analyzer (AATI) using the standard RNA sensitivity kit (DNF-471).

### NanoPARE library preparation

A detailed protocol can be found in Supplemental Methods. In brief, cDNA library preparation from 5 ng or less total RNA was carried out according to the original Smart-seq2 method (Picelli et al. 2013). cDNA was tagmented using the Illumina Nextera DNA library preparation kit, purified using the Zymo 5× DNA Clean and Concentrator kit (ZymoResearch), and eluted with nuclease-free water. For final enrichment PCR, the purified reaction was split and amplified either with Tn5.1/TSO enrichment oligonucleotide or Tn5.2/TSO enrichment oligonucleotide primer sets (Supplemental Table S1). PCR reaction products with Tn5.1/TSO enrichment oligonucleotide and Tn5.2/TSO enrichment oligonucleotide primer sets were pooled and purified using AMPureXP DNA beads.

For in vitro biochemical degradation of 5′ monophosphate-containing RNA, 100 ng of total RNA were treated with XRN1 exoribonuclease (NEB) for 60 min at 37°C in a 20-µL reaction volume containing 1× NEB Buffer 3 and 1 U of XRN1. The equivalent of 5 ng total RNA (1 µL of XRN1-treated reaction) was used for Smart-seq2 cDNA synthesis without additional purification.

### Next-generation sequencing

To control for library quality, final nanoPARE libraries were checked on an Agilent DNA HS Bioanalyzer Chip. Libraries with size ranges between 150 and 800 bp were diluted and sequenced to 10–15 million single-end 50-bp reads per sample using a custom sequencing primer (TSO_Seq) and a custom P5/P7 index primer mix on an Illumina HiSeq 2500 instrument (Supplemental Table S1; Supplemental Data S1).

### Classification of RNA 5′ ends

The nanoPARE analysis pipeline was written to identify capped and noncapped 5′ end features in the genome using paired nanoPARE (5P) reads Smart-seq2 (BODY) reads. Analysis is divided into four major steps:

1. Mapping of 5P and BODY reads to the genome (EndMap)
2. 5P end feature identification (EndGraph)
3. Classification of capped and noncapped 5P features (EndClass)
4. Transcript-level output of read noncapped reads (EndMask)

#### EndMap

FASTQ files were mapped to the *Arabidopsis thaliana* TAIR10 genome (Lamesch et al. 2012). EndMap first trims the appropriate adapter sequences using Cutadapt (Marcel 2011). To prevent reads with low sequence complexity from mapping to the genome, the I-complexity (Becher and Heiber 2012) of each FASTQ read was calculated*,* and reads with a per-nucleotide I-complexity <0.15 were removed. The remaining reads were then aligned to the genome with STAR (Dobin et al. 2013). Mapping behavior differs slightly between BODY and 5P libraries. All reads were mapped using the STAR settings:

--alignIntronMax 10000; --alignMatesGapMax 11000; --alignSJDBoverhangMin 1; --alignSJoverhangMin 10 --outFilterMismatchNmax 2; --outFilterMismatchNoverLmax .05; --outFilterMultimapNmax 100; --outSAMprimaryFlag AllBestScore; --outSAMtype BAM Unsorted

BODY reads were mapped with the additional settings:

--alignEndsType EndToEnd

5P reads were mapped with the additional settings:

--alignEndsType Local; --outFilterMatchNminOverLread 0.9

After alignment to the genome, a bias correction algorithm was applied to the aligned BAM file to adjust for sequence-specific biases in the BODY and 5P libraries. The bias correction method defined by Wang et al. (2017) was used, with two modifications to make it suitable for RNA rather than DNA data: (1) Only reads within exons of annotated genes were used to calculate the *k*-mer frequency matrix, and (2) the read depth for all positions with >1 read was set to 1, because RNA seq reads are not expected to have even coverage at all genomic locations. After bias correction, reads that mapped to more than one genomic location were assigned via a “rich-get-richer” algorithm similar to that employed by the software MuMRescue and MuMRescueLite (Faulkner et al. 2008; Hashimoto et al. 2009). First, coverage depth of uniquely mapping reads is calculated for each position in the genome. Multimappers are then binned by their mapping multiplicity (i.e., a read that maps to 10 locations in the genome has a multiplicity of 10). Beginning with a multiplicity of 2, all reads in that bin are sorted from lowest possible genomic position to highest, and each read is assigned in a multistep process: If ≥1 mapping position contains ≥1 existing read, the read is considered “unambiguous” and is assigned proportionally to its mapping locations using the formula $P_{i}=C_{i}/\sum_{\;j=1}^{n}C_{j}$, where *P*_*i*_ is the proportion of reads assigned to mapping location *i*, *C*_*i*_ is the total existing read coverage assigned to the genomic positions that comprise location *i*, and *n* is the number of mapping locations for the read. If the existing read coverage at all locations is 0, that read is not yet assigned. The process is repeated until no more unambiguous reads can be identified, then all remaining reads are assigned with equal weighting, or *P*_*i*_ = 1/*n*. This is repeated for multiplicities of 3–100. A bedGraph file of 5′ end counts is written for both strands of the genome. For 5P libraries, all nucleotides softclipped from the 5′ end of reads are stored as upstream untemplated nucleotides (uuNs).

#### EndGraph

Discrete 5P features were identified genome-wide via subtractive kernel density estimations. bedGraph files output from EndMap corresponding to a sample's 5P and BODY libraries were evaluated together. First, strand invasion artifacts (Tang et al. 2013) were masked based on complementarity to the last four bases of the template switching oligo, allowing up to one mismatch. Then, a scaling factor (*S*) was estimated to normalize the read depth of the 5P library against the BODY library using the formula

$$S=\frac{2F*10^{6}}{\sum_{i=1}^{n}(TPM_{i}*L_{i})}*\frac{R_{B}}{R_{E}},$$

where *n* is the total number of transcripts, *TPM*_*i*_ is the abundance of a transcript in transcripts per million, *L*_*i*_ is the length of a transcript in nucleotides, *F* is the mean fragment length of the BODY library, *R*_*B*_ is the total number of mapped BODY reads, and *R*_*E*_ is the total number of mapped 5P reads. Then, a Laplace kernel with a bandwidth of 15 nt was fit over the set of values (ER * *S*) – BR, where ER is the set of 5P end read counts and BR is the set of body read counts. Regions of continuous positive density were extracted and written as discrete features to a bed file.

#### EndClass

If a 5P experiment was designed with multiple replicates, EndClass merged all 5P features that could be reproducibly identified in ≥2 replicates. Then, the presence of a m7G cap was predicted for each replicable feature by calculating the proportion of reads containing upstream untemplated guanosine (uuG). A feature was considered capped if ≥10% of all reads from a sample type that map within the feature contained uuG; otherwise, the feature was considered noncapped.

#### EndMask

EndMask prepared a bedGraph file of 5P read positions relative to the start site of the dominant isoform of each gene in the reference annotation. Dominant isoforms were defined as the transcript isoform containing the most mapped reads. For nanoPARE libraries, this transcript-level bedGraph was generated with a cap-masked input in which 5P reads contained within replicable capped 5P features were discarded.

### Detection of sRNA-mediated cleavage sites with EndCut

Sequences of miRNAs and tasiRNAs annotated in TAIR10 or miRBase21 (Lamesch et al. 2012; Kozomara and Griffiths-Jones 2014) were selected (i.e., anno.mir.tas.fa) and randomized 1000 times each by the Python script sRNA_shuffler.py to produce anno.mir.tas.*i*.fa files, where *i* is an integer between 0 and 999. For annotated miRNA, tasiRNA and the corresponding 1000 randomized variants for each miRNA/tasiRNA, GSTAr.pl (https://github.com/MikeAxtell/GSTAr) was used to predict target sites in transcript models annotated as protein-coding genes, transposable element genes, or other RNAs (i.e., TAIR10_pc_teg_other_transcripts.fasta). Target sites were determined based on the level of complementarity between sRNAs and transcripts computed using previously developed criteria based on the frequency and position of the miRNA-target duplex mismatches (i.e., Allen scores) (Allen et al. 2005). As described above, nanoPARE data were processed by EndMask to exclude capped regions of transcripts from further analyses. Publicly available PARE data sets were downloaded from the Sequence Read Archive (NCBI) (Supplementary Data S1), but alignments overlapping capped features were not excluded from downstream analyses.

Predicted target sites and EndGraph output were used by EndCut_step1.sh to quantify the number of reads at predicted target sites and in adjacent 20-nt or 50-nt regions on the sense strand of the same transcript. Adjacent sites within 1 nt of predicted cleavage sites were not considered in order to not penalize sites for sRNA isoforms with slightly offset target recognition sites. The local enrichment of nanoPARE read 5′ ends at predicted cleavage sites relative to surrounding transcribed regions, or fold changes, were calculated by dividing the numbers of nanoPARE read 5′ ends at predicted cleavage sites +1 by the maximum numbers of reads in adjacent transcript regions +1. Allen scores were also assigned to each predicted cleavage site detected. For each randomized sRNA control set, EndCut_step2.R computed empirical cumulative distribution functions of fold changes (*ECDF*_*FC*_) and Allen scores (*ECDF*_*AS*_). These were then used as null models to test whether the observed cleavage site fold changes were not equal to or lesser than *ECDF*_*FC*_, as well as if the observed site Allen scores were not equal to or greater than *ECDF*_*AS*_. Final *P*-values were computed for each site by combining these two *P*-values using Fisher's combined probability test and then adjusted for multiple testing using the Benjamini-Hochberg method. For our analyses, we defined significant cleavage sites that had adjusted *P*-values < 0.05, fold changes > 1.0, and that were also represented by at least one read per 10 million transcriptome-mapping reads.

## Data access

All sequencing data generated in this study have been submitted to the NCBI Gene Expression Omnibus (GEO; https://www.ncbi.nlm.nih.gov/geo/) under accession number GSE112869. All software code is publicly available at GitHub (https://github.com/Gregor-Mendel-Institute/NanoPARE) and is available as Supplemental Code S1.

## Competing interest statement

M.A.S., M.J.K., and M.D.N. were employed by the Gregor Mendel Institute, a nonprofit research organization that has filed a patent application on the nanoPARE technology described in this work.

## Supplementary Material

Supplemental Material
