# Supplementary material for: NanoPARE: parallel analysis of RNA 5′ ends from low-input RNA
Source: Genome Res. 2018 Dec;28(12):1931–42. doi: 10.1101/gr.239202.118 (PMC6280765; doi:10.1101/gr.239202.118)
Supplement: Supplemental Material [file supp_28_12_1931__index.html]

NanoPARE: parallel analysis of RNA 5′ ends from low-input RNA — Supplemental Material 

# NanoPARE: parallel analysis of RNA 5′ ends from low-input RNA

## Supplemental Material

- Supplemental\_Data\_S2.xlsx
- Supplemental\_Data\_S4.xlsx
- Supplemental\_Data\_S5.xlsx
- Supplemental\_Data\_S6.xlsx
- Supplemental\_Data\_S7.xlsx
- Supplemental\_Data\_S8.xlsx
- Supplemental\_Code\_S1.zip
- Supplemental\_Data\_S1.xlsx
- Supplemental\_Data\_S3.xlsx
- Supplemental\_Material.pdf
