# Supplementary figures and images for: NanoPARE: parallel analysis of RNA 5′ ends from low-input RNA
Source: Genome Res. 2018 Dec;28(12):1931–42. doi: 10.1101/gr.239202.118 (PMC6280765; doi:10.1101/gr.239202.118)

miRNA slice site RPTM + 1 (log10)

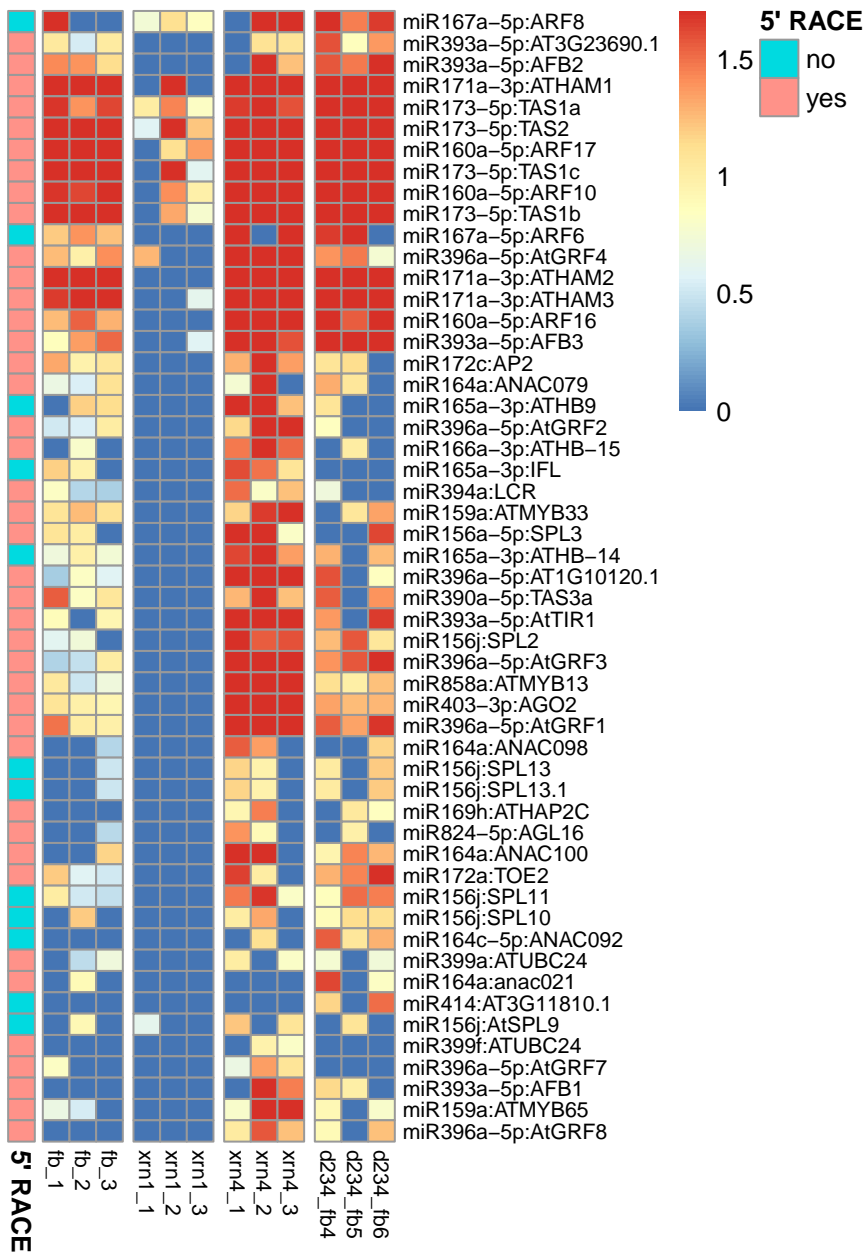

Supplement: Supplemental Material [file supp_gr.239202.118_Supplemental_Code_S1.zip › supplemental_code_S1/analysis_pipeline/EndCut.figures/resources/figures/fig.4g.miRNA.rpm.heatmap.pdf]

**tasiRNA slice site RPTM + 1 (log10)**

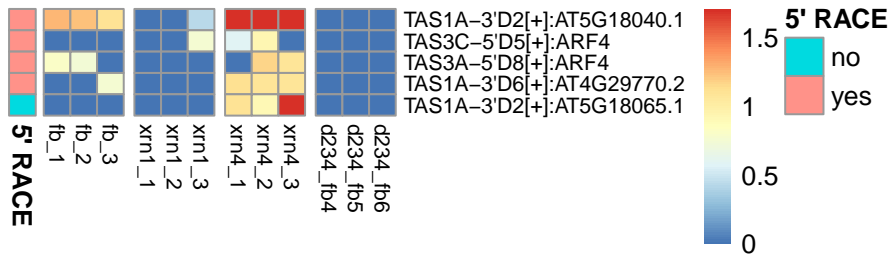

Supplement: Supplemental Material [file supp_gr.239202.118_Supplemental_Code_S1.zip › supplemental_code_S1/analysis_pipeline/EndCut.figures/resources/figures/fig.4g.tasiRNA.rpm.heatmap.pdf]
