# Supplementary material for: NanoPARE: parallel analysis of RNA 5′ ends from low-input RNA
Source: Genome Res. 2018 Dec;28(12):1931–42. doi: 10.1101/gr.239202.118 (PMC6280765; doi:10.1101/gr.239202.118)
Supplement: Supplemental Material [file supp_gr.239202.118_Supplemental_Material.pdf]

Supplemental Material for "NanoPARE: Parallel analysis of RNA 5′ ends from low input RNA" by Schon, Kellner et al.

Table of Contents

Table/Figure	*Page*
**Supplemental Tables**	
Table S1. Oligonucleotides used in this publication	2
**Supplemental Figures**	
Figure S1. Untemplated upstream nucleotide content	3
Figure S2. Genomic distribution of capped and noncapped features	4
Figure S3. IGV browser tracks with examples of RNA pol II transcripts	5
Figure S4. IGV browser tracks with examples of non-pol II transcripts	6
Figure S5. Distribution of capped and noncapped features by gene type	7
Figure S6. Noncoding RNA 5′ ends detected by nanoPARE	8
Figure S7. Global changes to 5′ feature abundance in *xrn4-5* mutant floral buds	9
Figure S8. Detection of predicted miRNA-mediated cleavage sites	10
Figure S9. IGV browser tracks with examples of small RNA cleavage sites	11
Figure S10. EndCut workflow	12
Figure S11. Variation of small RNA-target cleavage activity	13
**Supplemental Methods**	
NanoPARE Protocol	14

Supplemental Data Files S1-S8 are provided separately as Excel spreadsheets.

- Data S1. Summary of high-throughput sequencing generated or reanalyzed for this study
- Data S2. Floral bud 5′ end features identified by EndGraph
- Data S3. EndGraph features associated with noncoding RNAs
- Data S4. EndCut analysis of microRNA cleavage sites in nanoPARE data
- Data S5. EndCut analysis of tasiRNA cleavage sites in nanoPARE data
- Data S6. Published microRNA target interactions validated by 5′ RACE
- Data S7. EndCut analysis of microRNA cleavage sites in public degradome data
- Data S8. EndCut analysis of tasiRNA cleavage sites in public degradome data

Supplemental Code S1. Scripts used to generate figures and datasets in this study.

Table S1. Oligonucleotides used in this publication

Name	Sequence	step used	additional information
Anchored oligo-dT primer	AAGCAGTGGTATCAACGCAGAGTACT30VN	Reverse transcription	' N' is any base and 'V' is either 'A', 'C' or 'G'
TSO	AAGCAGTGGTATCAACGCAGAGTACrGrG+G	Reverse transcription	'+' is LNA base, 'r' is ribonucleotide, HPLC purified
TSO 5' Biotin	/5Biosg/AAGCAGTGGTATCAACGCAGAGTACrGrG+G	Reverse transcription	5'-Biotinylated, '+' is LNA base, 'r' is ribonucleotide, HPLC purified, prefered to avoid concatemerization
ISPCR	AAGCAGTGGTATCAACGCAGAGT	PCR-preamplification	
P5_TSO_N501	AATGATACGGCGACCACCGAGATCTACACTAGATCGCCTAGCAAGCAGTGGTATCAACGCAGAGTACGGG	NanoPARE 5'end enrichment	Illumina Nextera N501 index, PAGE purified
P5_TSO_N502	AATGATACGGCGACCACCGAGATCTACACCTCTCTATCTAGCAAGCAGTGGTATCAACGCAGAGTACGGG	NanoPARE 5'end enrichment	Illumina Nextera N502 index, PAGE purified
P5_TSO_N503	AATGATACGGCGACCACCGAGATCTACACTATCCTCTCTAGCAAGCAGTGGTATCAACGCAGAGTACGGG	NanoPARE 5'end enrichment	Illumina Nextera N503 index, PAGE purified
P5_TSO_N504	AATGATACGGCGACCACCGAGATCTACACAGAGTAGACTAGCAAGCAGTGGTATCAACGCAGAGTACGGG	NanoPARE 5'end enrichment	Illumina Nextera N504 index, PAGE purified
P5_TSO_N505	AATGATACGGCGACCACCGAGATCTACACGTAAGGAGCTAGCAAGCAGTGGTATCAACGCAGAGTACGGG	NanoPARE 5'end enrichment	Illumina Nextera N505 index, PAGE purified
P5_TSO_N506	AATGATACGGCGACCACCGAGATCTACACACTGCATACTAGCAAGCAGTGGTATCAACGCAGAGTACGGG	NanoPARE 5'end enrichment	Illumina Nextera N506 index, PAGE purified
P5_TSO_N507	AATGATACGGCGACCACCGAGATCTACACAAGGAGTACTAGCAAGCAGTGGTATCAACGCAGAGTACGGG	NanoPARE 5'end enrichment	Illumina Nextera N507 index, PAGE purified
P5_TSO_N508	AATGATACGGCGACCACCGAGATCTACACCTAAGCCTCTAGCAAGCAGTGGTATCAACGCAGAGTACGGG	NanoPARE 5'end enrichment	Illumina Nextera N508 index, PAGE purified
P5_TSO_S510	AATGATACGGCGACCACCGAGATCTACACCGTCTAATCTAGCAAGCAGTGGTATCAACGCAGAGTACGGG	NanoPARE 5'end enrichment	Illumina Nextera S510 index, PAGE purified
P7_Tn5.1_N701	CAAGCAGAAGACGGCATACGAGATTAAGGCGATCGTCGGCAGCGTC	NanoPARE 5'end enrichment	Illumina Nextera N701 index, PAGE purified
P7_Tn5.2_N701	CAAGCAGAAGACGGCATACGAGATTAAGGCGAGTCTCGTGGGCTCGG	NanoPARE 5'end enrichment	Illumina Nextera N701 index, PAGE purified
TSO_seq_read1	CTAGCAAGCAGTGGTATCAACGCAGAGTACGGG	Illumina custom sequencing primer for i5 index	Custom sequencing primer

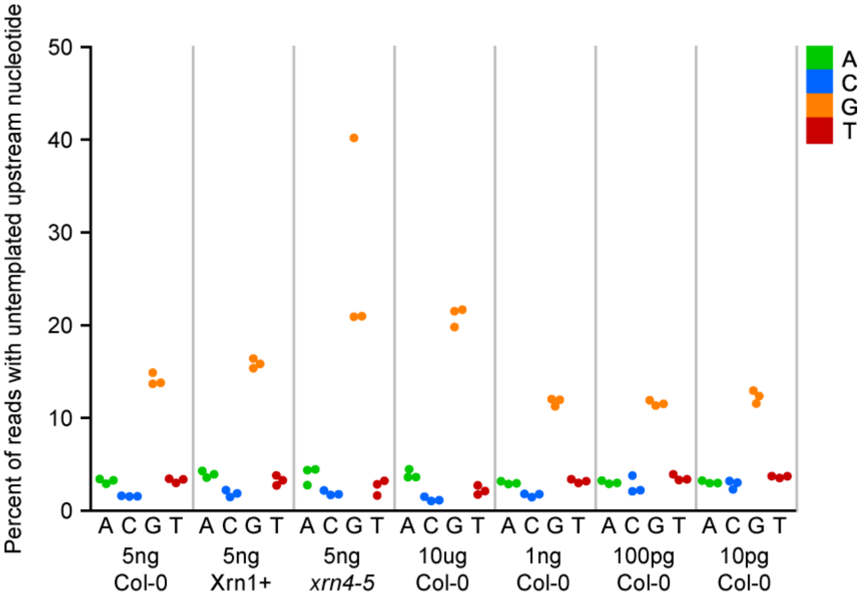

Supplemental Figure S1. Untemplated upstream nucleotide content. Proportion of genome-matching nanoPARE reads with untemplated upstream nucleotides (uuNs) in floral bud samples.

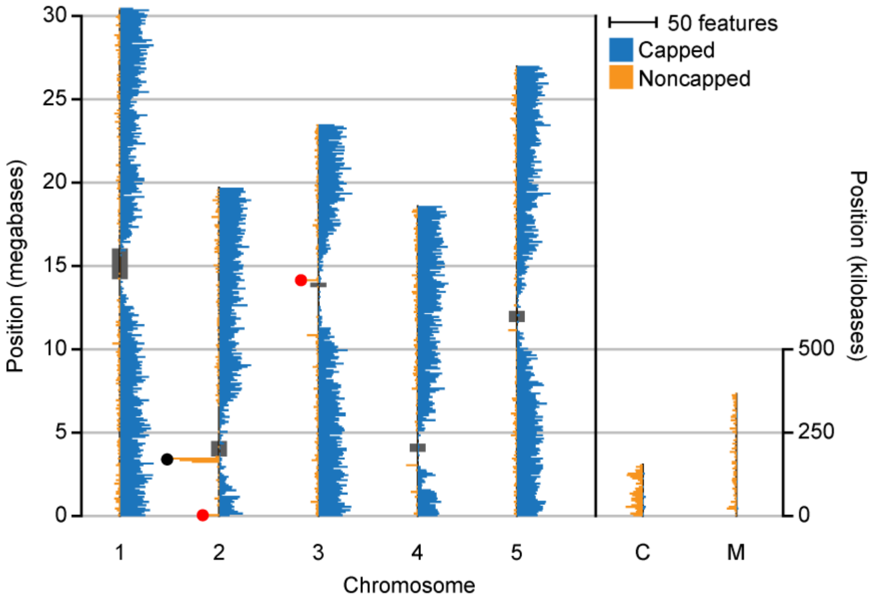

Supplemental Figure S2. Genomic distribution of capped and noncapped features. Barplots depicting the number of capped (blue) and noncapped (orange) features contained in bins across the genome. A bin size of 100 kilobases was used for nuclear chromosomes, and a bin size of 5 kilobases was used for mitochondrial and chloroplast genomes. Centromeres are marked by gray rectangles. Red circles are centered over the two 45S ribosomal RNA loci in the TAIR10 genome assembly on the short arms of chromosomes 2 and 3, and the black circle on chromosome 2 marks an insertion of the mitochondrial genome into the pericentromeric region.

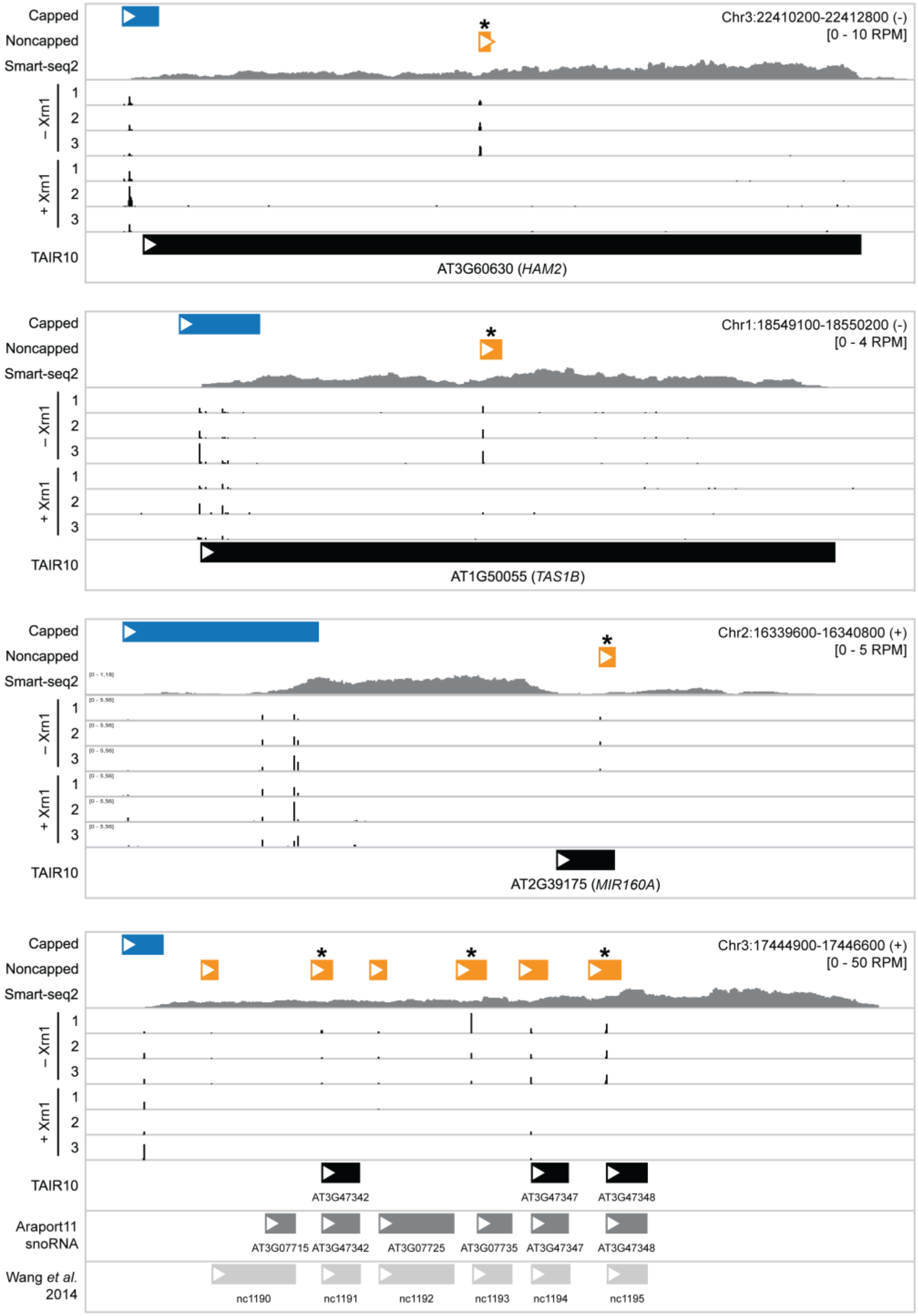

Supplemental Figure S3. IGV browser tracks with examples of RNA pol II transcripts. Blue boxes depict capped features, orange boxes depict noncapped features, and black boxes depict TAIR10 exons. Triangles show the feature orientation, and an asterisk marks features that are significantly less abundant in Xrn1+ samples (*P*-values < 0.05, DEseq2). (*A*) Protein coding mRNA HAM2. (*B*) Long noncoding RNA TAS1B. (*C*) Primary microRNA MIR160A. (*D*) Unannotated primary snoRNA transcript containing six mature snoRNAs.

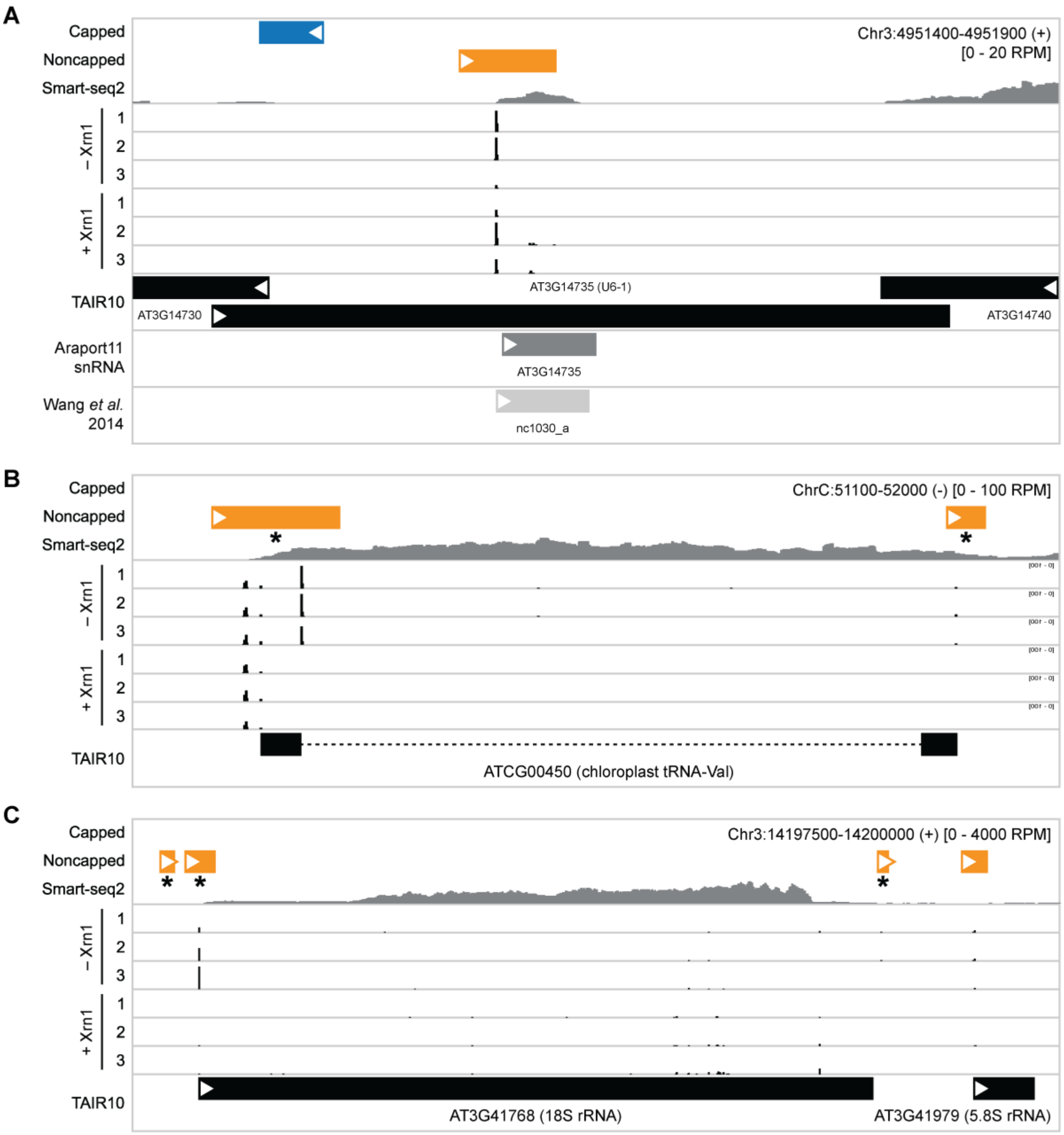

Supplemental Figure S4. IGV browser tracks with examples of non-pol II transcripts. Tracks are formatted as in Supplemental Figure 3. (*A*) Small nuclear RNA U6-1. (*B*) Chloroplast group-II intron containing primary tRNA-Val. (*C*) 18S and 5.8S ribosomal RNA.

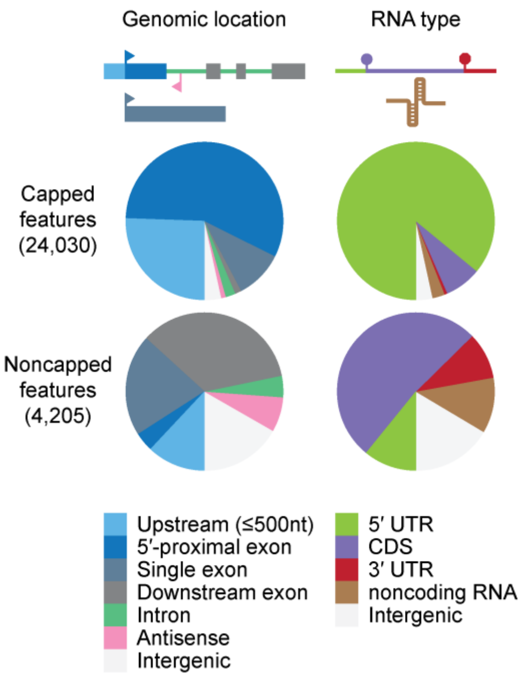

Supplemental Figure S5. Distribution of capped and noncapped features by gene type. Pie charts grouping all capped features (*top*) and noncapped features (*bottom*) by their location relative to TAIR10 gene annotations based on their genomic location (*left*) and the type of RNA (*right*).

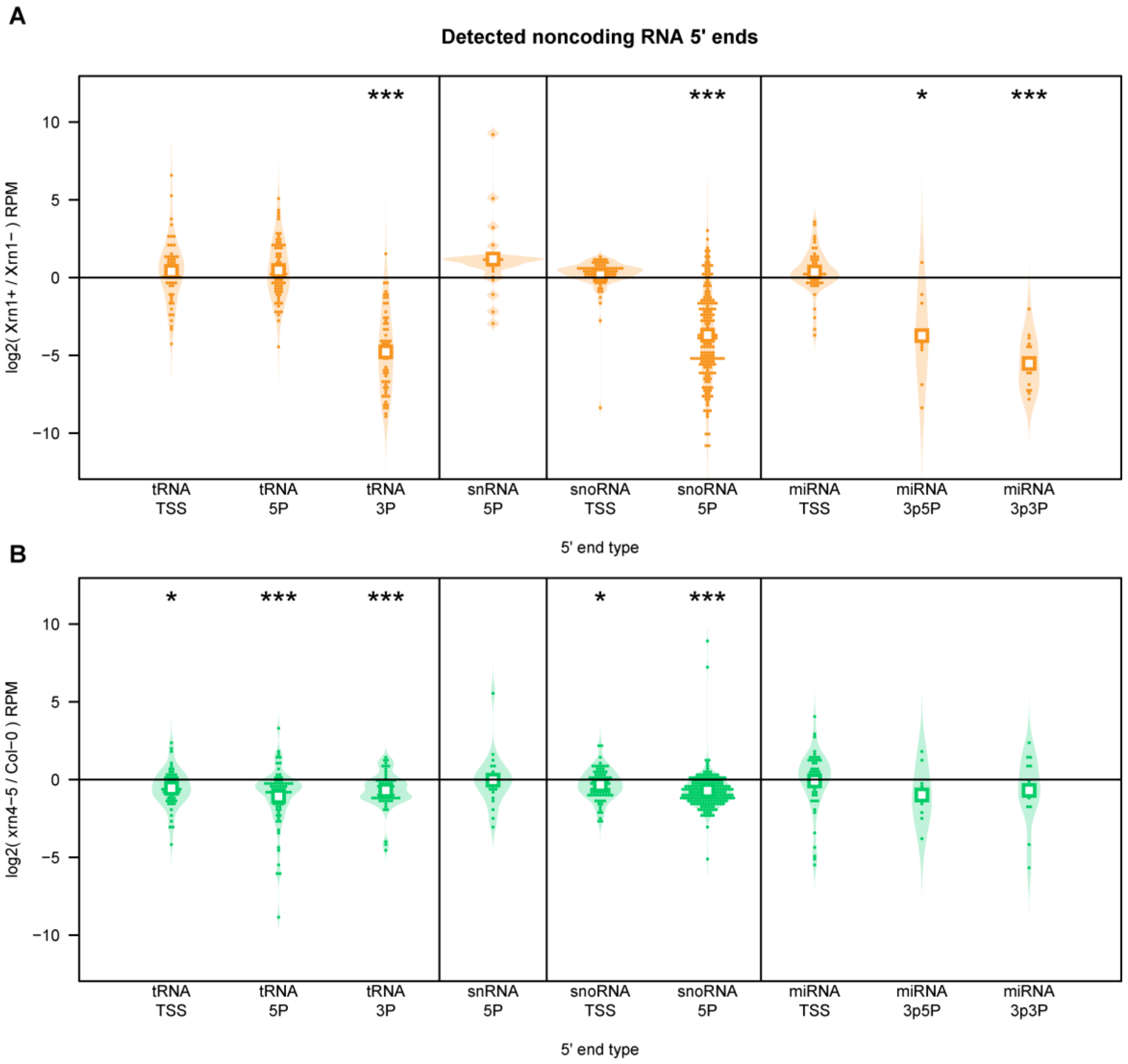

Supplemental Figure S6. Noncoding RNA 5′ ends detected by nanoPARE. Stripcharts of all RNA 5′ ends identified by EndGraph from non-protein coding transcripts showing fold change relative to untreated Col-0 nanoPARE reads. (*A*) Log_2 fold change after treatment with Xrn1 enzyme. (*B*) Log_2 fold change in *xrn4-5* mutant floral buds. * and *** indicate P-values < 0.05 and 0.001, respectively, based on two-tailed Student's t-test.

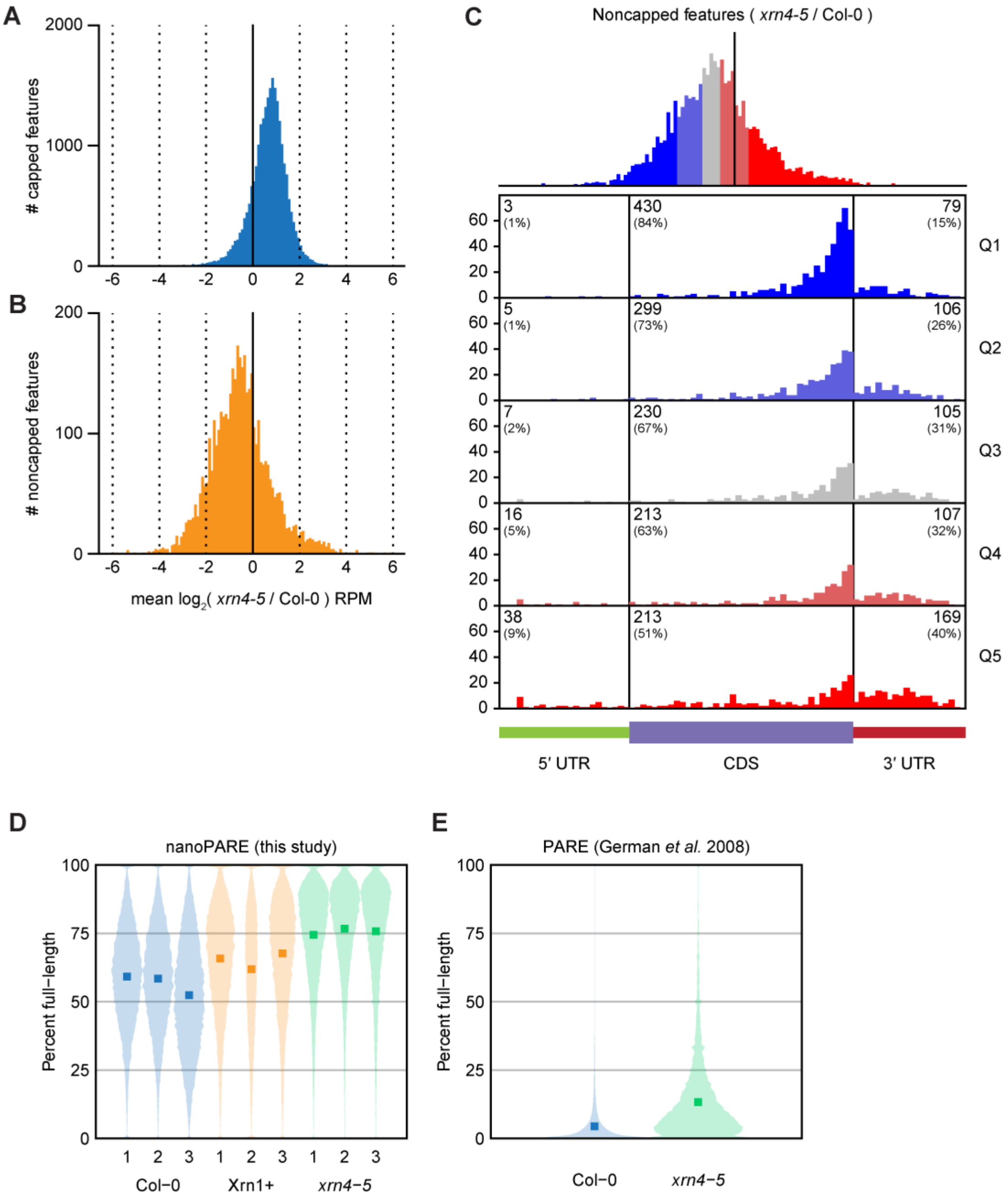

Supplemental Figure S7. Global changes to 5′ feature abundance in *xrn4-5* mutant floral buds. (*A*) Log_2 fold change of capped feature RPM. (*B*) Log_2 fold change noncapped feature RPM. (*C*) mRNA metaplots as in Figure 2E for noncapped features grouped into quintiles based on change in abundance in *xrn4-5*. (*D*) Violin plots showing the percent of nanoPARE reads contained within a gene's capped feature(s) for all genes where at least one capped feature could be identified. Squares mark the mean proportion of full-length reads across all genes. (*E*) Analysis of German et al. 2008 PARE data using the same criteria as in D.

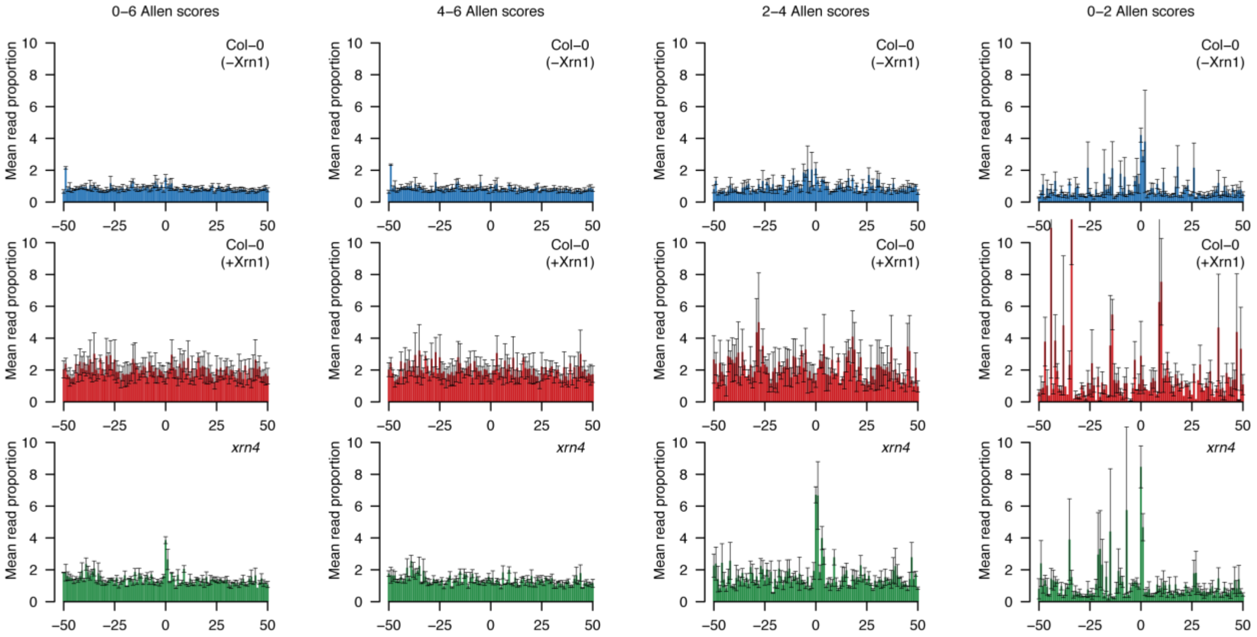

Supplemental Figure S8. Detection of predicted miRNA-mediated cleavage sites. Number of nanoPARE read 5′ ends mapping within 50 nt of all predicted miRNA cleavage sites are shown as a percentage of the total number of nanoPARE reads detected for each transcript. All predicted sites (0 – 6 Allen scores) and predicted sites with low (4 – 6 Allen scores), medium (2 – 4) or high (0 – 2 Allen scores) miRNA-target duplex complementarities are shown from *left* to *right*.

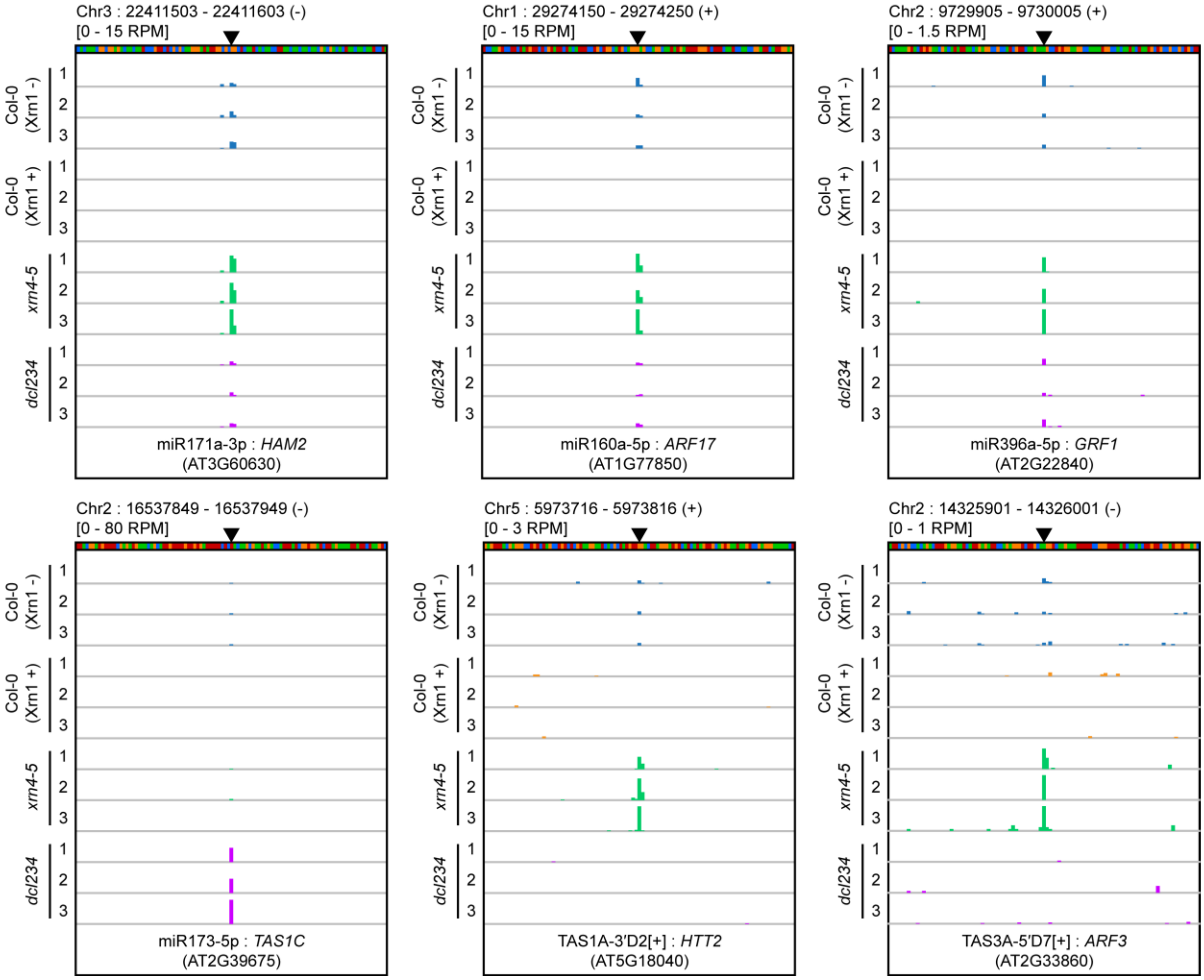

Supplemental Figure S9. IGV browser tracks with examples of small RNA cleavage sites. All tracks in each panel are normalized by the number of genome-matching reads per million (RPM) and were scaled by the values stated above the panels. Expected cleavage position of the listed small RNA species is marked by a black triangle. Nucleotide sequence is depicted by colored bars above each panel: green – A, blue – C, orange – G, red – T.

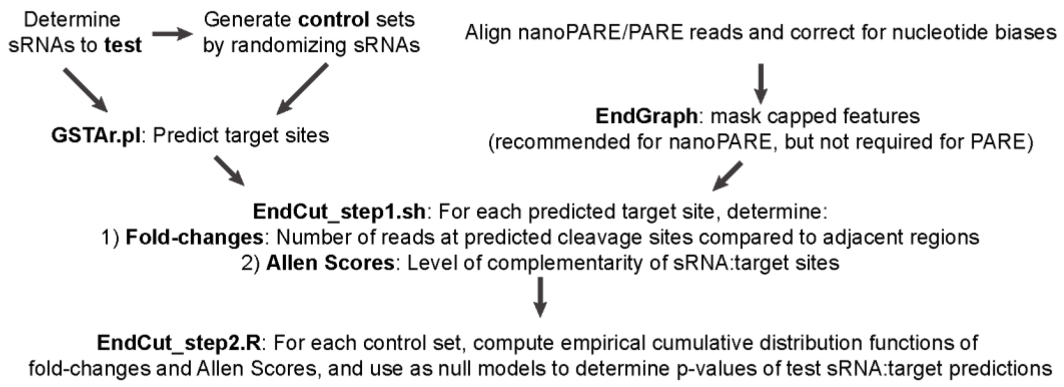

Supplemental Figure S10. Diagram of EndCut workflow used to detect sRNA-mediated cleavage sites. See Methods for details.

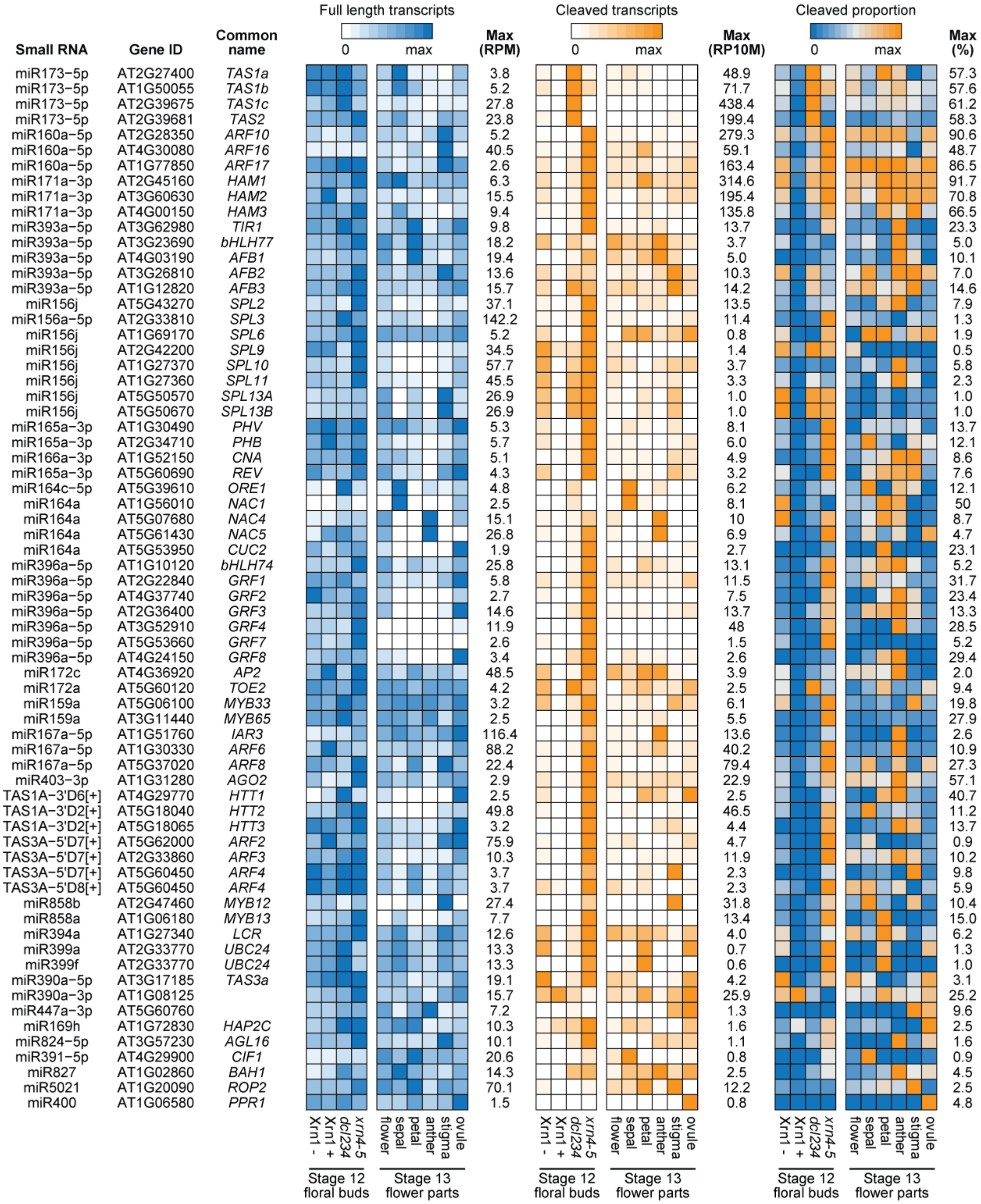

Supplemental Figure S11. Heatmaps of mean capped feature RPM (*left*), cleaved transcript RP10M (*center*), and percent cleaved of capped and cleaved RPM (*right*) for all sRNA-target interactions identified in at least two bioreplicates of one or more sample types (i.e. high-confidence interactions). Each row is scaled to the maximum value measured for that row, displayed to the right of each heatmap.

Supplemental Methods

NanoPARE Protocol

The initial part of this protocol (Reverse Transcription through Tagmentation) is directly from a Smart-seq2 protocol (Picelli et al, 2013). We have included it here for convenience and compatibilities with the nanoPARE protocol.

Materials

Oligonucleotides
- see Table S1

Reverse Transcription
- dNTP mix (10 mM; ThermoFisher)
- Superscript II reverse transcriptase (200 U/µL; Invitrogen/Life Tech)
- Superscript II First-Strand Buffer (5×; Invitrogen/Life Tech)
- DTT (100 mM; Invitrogen/Life Tech)
- Murine RNAse inhibitor (40 U/µL; NEB)
- Betaine (5 M; Sigma)
- $MgCl_2$ (100 mM; Sigma)

PCR preamplification

- KAPA HiFi HotStart ReadyMix (2×; KAPPA Biosystems)
- EB solution (Qiagen) or Nuclease free water
- High-sensitivity DNA chip (Agilent Bioanalyzer)

Tagmentation reaction and final PCR
- Nextera DNA Sample Preparation kit (Illumina)

Procedure

Reverse transcription

1. Prepare first-strand reaction mix as follows (7 µL per reaction):

   0.5 µL Superscript II RT
   0.25 µL Murine RNase Inhibitor
   2 µL Superscript II first-strand buffer (5×)
   0.25 µL DTT (0.1 M)
   2 µL Betaine (5 M)
   0.9 µL $MgCl_2$ (100 mM)
   1 µL TSO oligo (10 µM)
   0.1 µL Nuclease free water
   -----
   7 µL total

2. Mix 1 µL of total RNA with 1 µL of anchored oligo-dT primer (10 µM) and 1 µL of dNTP mix.
3. Denature at 72 ˚C for 3 minutes and place immediately on ice.

4. Add 7 µL of first-strand reaction mix to above RNA/oligo-dT/dNTP mix, and mix by pipetting up and down 10 times.
5. In thermal cycler incubate at 42 ˚C for 90 minutes, then 10 cycles of (50 ˚C for 2 minutes, 42 ˚C for 2 minutes) and finally inactivate by incubating at 70 ˚C for 15 minutes.

<u>PCR preamplification</u>

1. Make PCR mastermix, add directly to inactivated RT reaction above, and mix by pipetting up and down 10 times.

   14 µL Nuclease-free water
   25 µL 2× KAPA HiFi HotStart ReadyMix
   1 µL 10 µM ISPCR Primer (1 uM final)
   10 µL RT reaction from above

   50µL total

2. In thermal cycler incubate at 98 ˚C for 3 minutes, 11-20 cycles of (98 ˚C for 15 seconds, 67 ˚C for 20 seconds, 72 ˚C for 6 minutes) and final extension at 72 ˚C for 5 minutes.

   Note: Adjust PCR preamplification cycle count for RNA input. Here are some guidelines for total RNA: 11 cycles for 5 ng, 13 cycles for 1 ng, 15-16 cycles for 100 pg and 16-18 cycles for 10 pg. We recommend performing negative controls by replacing the RT reaction products with water and using the maximum number of PCR cycles used for samples.

3. Purify PCR product using a 1:1 ratio of AMPure XP beads
   a. Let beads come to room temperature for 30 minutes.
   b. Vortex two times for 2 seconds each and add 50 µL of beads to each sample.
   c. Pipette up and down 10 times and incubate at room temperature for 8 minutes.
   d. Place on magnetic stand for five minutes.
   e. Pipette off supernatant and try not to disturb the beads.
   f. Keep on magnetic stand and add 200 µL of fresh 80% ethanol, wait 30 seconds and remove supernatant.
   g. Repeat washing step and remove supernatant completely. Note: Tn5 is sensitive to ethanol.
   h. Incubate at room temperature for five minutes, and remove from magnetic stand.
   i. Add 16 µL of elution buffer (EB solution from Qiagen), pipette up and down 10 times, put on magnetic stand for 2 minutes and collect 15 µL in new Eppendorf tube (1 µL is left to avoid taking up beads).

4. Run 1 µL on High-sensitivity Bioanalyzer DNA chip (Agilent) or Fragment Analyzer (Advanced Analytical) to determine median size and range. Note: expected sizes vary by organism, but fragments < 300 bp should be negligible (Supplemental Methods Fig. 1). Measure concentration between 300 and 9000 bp.

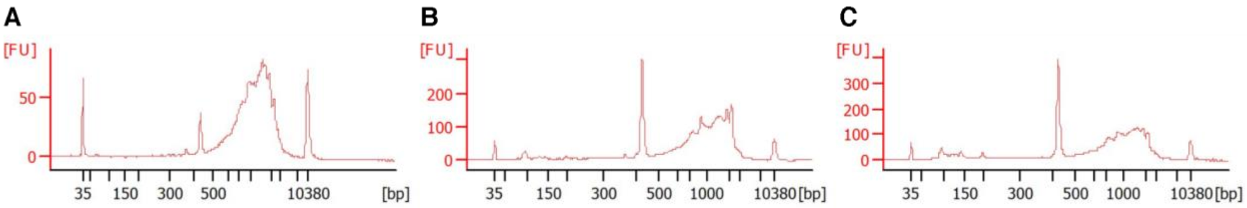

Supplemental Methods Figure 1. Representative profiles of cDNA generated from (*A*) 1 ng of total RNA input and 13 PCR cycles, (*B*) 100 pg of total RNA input and 15 PCR cycles and (*C*) 10 pg of total RNA input and 18 PCR cycles.

Note: This is a good pausing point in the protocol. cDNA can be frozen and stored at -20 ˚C.

<u>Tagmentation using Nextera DNA Library preparation kit</u>

- Use 5 ng of cDNA for tagmentation

  x µL cDNA (Final 5 ng)
  2.5 µL Tn5 Tagmentation enzyme
  12.5 µL Tagmentation Buffer
  x µL Nuclease free water to 25 µL
  ----
  25 µL Total

- Incubate for 5 min at 55 ˚C and cool to 4 ˚C or place on ice (Tn5 is active at room temperature and overtagmentation can occur)

- Clean up with DNA Clean & Concentrator-5 kit (Zymo Research)
  - Add 125 µL binding solution to 25 µL tagmentation reaction
  - Apply to column and spin for 30 seconds at >12000xg
  - Wash 2× with 200 µL washing buffer
  - Elute in 21 µL of resuspension buffer

<u>5'-end enrichment PCR (NanoPARE)</u>

At this point, transcript body regions can be enriched from the above PCR amplification products using standard Nextera primers according to Picelli et al. (i.e. Smart-seq2; Picelli et al. 2013) or RNA 5′ ends can be enriched as described below.

- Split tagmentation reaction in two 10 µL aliquots and perform enrichment PCR with either Tn5.1 or Tn5.2 as the reverse primer and P5 N5xx TSO enrichment primer as the forward primer

  7.5 µL Nextera PCR Mastermix
  2.5 µL 10µM P5 N5xx TSO enrichment Primer (1 uM final)
  2.5 µL 10µM P7 Tn5.1/Tn5.2 enrichment Primer (1 uM final)
  10 µL Tagmentation reaction
  2.5 µL Nuclease free water
  -----
  25 µL total

- In thermal cycler perform enrichment PCR by incubating at 72 ˚C for 3 minutes to allow extension of single-stranded DNA adapters to form double-stranded DNA), 98 ˚C for initial denature, then 10 cycles of (98 ˚C for 10 seconds, 63 ˚C for 30 seconds, 72 ˚C for 3 minutes), inactivate by incubating at 72 ˚C for 1 minute and hold at 12 ˚C.

- Pool both 25 µL reactions and clean up using AMPure XP beads in 1:1 ratio as described above (50 µL beads)

<u>Quality control</u>

- Run High-sensitivity Bioanalyzer DNA chip (Agilent) or Fragment Analyzer (Advanced Analytical) to check for mean fragment length. Informative fragments should have a length of 150-800 bp. Overtagmentation results in short fragments of about 135 bp long and which will have low mappability (Supplemental Methods Figure 2). In such cases, check cDNA input and make sure that it does not exceed 5ng for 2.5 µL Tn5 Enzyme mix. Also avoid keeping tagmentation reaction for too long on thermoblock.

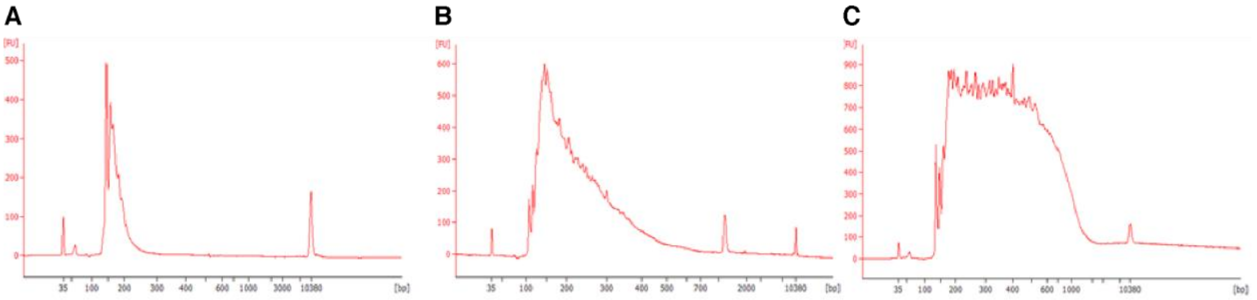

Supplemental Methods Figure 2. Representative profiles of nanoPARE libraries where tagmentation reaction was performed either (*A*) excessively (i.e. overtagmented), (*B*) optimally or (*C*) insufficiently (i.e. undertagmented).

<u>Next-generation sequencing</u>

- Quantify library and pool in equimolar ratio (about 4 nM each. Follow guidelines from Illumina Platform).
- For Illumina HiSeq2500 machines (this study): Sequence with custom sequencing primer listed in Supplemental Table S1 (TSO_seq_read1). Typically Single Read 50 base mode at a sequencing depth >10-15 million reads is sufficient. If possible, replace Index 1 (i7) Read primer with an equimolar mix of primers P7_Tn5.1_N701 and P7_Tn5.1_N701 to allow for dual indexing. Alternatively, run dual indexing with chemistry-only cycles for Read 1 (i7) and use i5 sequences only for indexing.
- For Illumina NextSeq machines: Replace Index 2 (i5) Read primer with a custom primer (CCCGTACTCTGCGTTGATACCACTGCTTGCTAG), which is the reverse complement of custom sequencing primer listed in Supplemental Table S1 (TSO_seq_read1).
